# From germline genome to highly fragmented somatic genome: genome-wide DNA rearrangement during the sexual process in ciliated protists

**DOI:** 10.1007/s42995-023-00213-x

**Published:** 2024-02-12

**Authors:** Liping Lyu, Xue Zhang, Yunyi Gao, Tengteng Zhang, Jinyu Fu, Naomi A. Stover, Feng Gao

**Affiliations:** 1 Key Laboratory of Evolution & Marine Biodiversity (Ministry of Education), and Institute of Evolution & Marine Biodiversity, Ocean University of China, Qingdao, 266003 China; 2 Department of Biology, Bradley University, Peoria, IL 61625 USA; 3 Laoshan Laboratory, Qingdao, 266237 China

**Keywords:** Alternative processing, Ciliates, Gene scrambling, Genome rearrangement, Germline genome, Somatic genome

## Abstract

Genomes are incredibly dynamic within diverse eukaryotes and programmed genome rearrangements (PGR) play important roles in generating genomic diversity. However, genomes and chromosomes in metazoans are usually large in size which prevents our understanding of the origin and evolution of PGR. To expand our knowledge of genomic diversity and the evolutionary origin of complex genome rearrangements, we focus on ciliated protists (ciliates). Ciliates are single-celled eukaryotes with highly fragmented somatic chromosomes and massively scrambled germline genomes. PGR in ciliates occurs extensively by removing massive amounts of repetitive and selfish DNA elements found in the silent germline genome during development of the somatic genome. We report the partial germline genomes of two spirotrich ciliate species, namely *Strombidium* cf. *sulcatum* and *Halteria grandinella*, along with the most compact and highly fragmented somatic genome for *S.* cf. *sulcatum*. We provide the first insights into the genome rearrangements of these two species and compare these features with those of other ciliates. Our analyses reveal: (1) DNA sequence loss through evolution and during PGR in *S*. cf. *sulcatum* has combined to produce the most compact and efficient nanochromosomes observed to date; (2) the compact, transcriptome-like somatic genome in both species results from extensive removal of a relatively large number of shorter germline-specific DNA sequences; (3) long chromosome breakage site motifs are duplicated and retained in the somatic genome, revealing a complex model of chromosome fragmentation in spirotrichs; (4) gene scrambling and alternative processing are found throughout the core spirotrichs, offering unique opportunities to increase genetic diversity and regulation in this group.

**Supplementary Information:**

The online version contains supplementary material available at 10.1007/s42995-023-00213-x.

## Introduction

For most eukaryotes, the genetic sequences received from parents are used directly by the offspring. However, in some species the germline and somatic genomes differ dramatically in their composition. For example, the germ cells of sea lampreys possess the full complement of genomic DNA, whereas the somatic cells possess a smaller fraction of the germline genome, which is the result of programmed genome rearrangement (PGR) (Smith et al. 2018; Timoshevskiy et al. 2016). PGR occurs from vertebrates to protists. Examples of PGR at a genome-wide level, where specific regions throughout the germline chromosomes are eliminated during development of the somatic genome, have been described in nematodes (Müller and Tobler 2000), copepods (Kloc and Zagrodzinska 2001), hagfish (Kubota et al. 1997), foraminiferans (Lee et al. 1991), and ciliates (Jahn and Klobutcher 2002).

Although PGR is widespread among distinct groups of eukaryotes, it appears most pronounced in ciliates, a highly divergent group of single-celled eukaryotes that is estimated to be about one billion years old (Arnaiz et al. 2012; Chen et al. 2014; Hamilton et al. 2016; Maurer-Alcalá et al. 2018a; Wang et al. 2022; Zhao et al. 2021). Ciliates display nuclear dimorphism, where separate nuclei contain the diploid germline genome (the micronucleus, or MIC) and the polyploid somatic genome (macronucleus, or MAC) within the same cell. The MAC is transcriptionally active throughout the entire life cycle and undergoes amitotic division during asexual reproduction (Katz 2001; Prescott 1994; Seyfert and Cleffmann 1982). In contrast, the transposon-rich MIC is transcriptionally silent in vegetative cells (Chen et al. 2014; Tian et al. 2022) and divides mitotically during vegetative (or asexual) growth (Prescott 1994). During the sexual process (conjugation), the MIC becomes transcriptionally active, undergoing meiotic and mitotic events to produce both migratory and stationary haploid gametic nuclei. Conjugating pairs exchange their migratory gametic nuclei which then fuse with the stationary gametic nucleus found in the partner cell to form a zygotic nucleus (Gong et al. 2020; Jiang et al. 2019; Zhang et al. 2022). The zygotic nucleus undergoes successive mitotic divisions followed by the development of new MACs and MICs. PGR happens specifically during development of the MAC, a series of events that results in chromosomal fragmentation, DNA elimination, and DNA amplification (Gao et al. 2023; Prescott 1994; Rzeszutek et al. 2020).

The extent and pattern of PGR vary dramatically among ciliates. *Tetrahymena thermophila* (class Oligohymenophorea) generates 181 MAC chromosomes from five zygotic chromosomes (Sheng et al. 2020; Wang et al. 2021), eliminating ~ 12,000 germline-limited sequences (called internal eliminated sequences, IES), which account for over 30% of its germline genome sequences (Fass et al. 2011; Hamilton et al. 2016). In comparison, ciliates in the class Spirotrichea (spirotrichs, the focus of this study) process their genomes much more extensively. *Oxytricha trifallax*, for example, generates ~ 19,000 “gene-sized” nanochromosomes from ~ 100 zygotic chromosomes, eliminating over 200,000 IESs (~ 90% of the germline genome) (Chen et al. 2014; Lindblad et al. 2019). The mean length of the resulting nanochromosomes is only 3.2 kb, with each containing a minimal amount of non-coding DNA outside the coding domains, untranslated regions, and telomeres. PGR in this species is also more complicated, with 20–30% of the macronucleus-destined sequences (MDS, the sequences retained in the somatic genome) “scrambled” in a non-canonical order in the germline genome, requiring complex genome rearrangements to form a functional MAC genome. Even for species in the same class, the patterns of PGR can be quite different. For example, the somatic genomes of the spirotrich genus *Euplotes* (e.g., *E. vannus* and *E. woodruffi*) have similar size and structure to, but larger MDSs than, *O. trifallax* (Chen et al. 2014, 2019; Feng et al. 2022). Furthermore, only about 4–7% of the genes in the germline genome are scrambled in *Euplotes*, far lower than the 20–30% seen in *Oxytricha*.

To expand our knowledge of the diversity of genome structures and the evolution of complex genome rearrangements, we focus on spirotrichs which are characterized by having germline genomes and highly fragmented somatic genomes with gene-sized ‘‘nanochromosomes”. In the present study, we provide the first insights into the structure of the germline genome of *Strombidium* cf. *sulcatum* and *Halteria grandinella. Strombidium* cf. *sulcatum* belongs to the subclass Oligotrichia and is one of the best known marine microzooplankton species as it plays important roles in food webs and energy flow in marine pelagic waters (Song et al. 2020; Wang et al. 2021). *Halteria grandinella* is a cosmopolitan species that lives predominantly in freshwater habitats and is typically planktonic. It was recently shown to be closely related to hypotrichs based on both phylogenetic and phylogenomic analyses (Gao et al. 2016; Wang et al. 2019), although it shares similar morphological features with oligotrichs which might result from convergent evolution to adapt to a planktonic lifestyle. We also report the first somatic genome of *S.* cf. *sulcatum* and compare its features with *H. grandinella* and two other model ciliates in the same class, namely *E. vannus* (subclass Euplotia) and *O. trifallax* (subclass Hypotrichia). Specifically, comparing the somatic and germline genome sequences in *S*. cf. *sulcatum* and *H. grandinella* allows us to compare the features of PGR in these species with those in *E. vannus* and *O. trifallax*. This comparison offers detailed insights into the diversity and architecture of PGR in ciliates with gene-sized somatic chromosomes.

## Materials and methods

### Cell isolation and nucleic acid extraction

*Halteria grandinella* was isolated from a freshwater pond in Baihuayuan Park (36°04ʹ N, 120°22ʹ E) in Qingdao, northern China. *Strombidium* cf. *sulcatum* was collected from surface seawater in Daya Bay (22°37ʹ N, 114°38ʹ E) in Huizhou, southern China. Species were identified by morphological features and small subunit ribosomal RNA gene sequences. Single cells of each species were isolated, washed, and cultivated at room temperature (ca. 25 °C) in flasks containing autoclaved habitat water. *Escherichia coli* strain DH5α was used as the food source. All downstream experiments were based on clones produced by asexual reproduction of a single cell. Cells were starved for 48 h before DNA or RNA extraction to reduce contamination by *E. coli* and other bacteria. Cells were harvested by centrifugation at 300 *g* for 5 min. DNA was extracted using phenol chloroform extraction followed by ethanol precipitation. RNA extraction was performed with the Rneasy Plus Mini Kit (Qiagen, Germany, Cat No. 74134). QIAGEN REPLI-g Single Cell Kit (Cat No. 150345) was used for MIC genome amplification from each single cell with Multiple Displacement Amplification (MDA). All operations followed the manufacturer’s instructions.

### High-throughput sequencing

DNA libraries were generated using Truseq Nano DNA HT Sample Preparation Kits (cat. FC-121-2003, Illumina USA) following the manufacturer’s recommendations. Briefly, the DNA sample was fragmented by sonication to a size of 350 bp. The DNA fragments were end-polished, A-tailed, and ligated with the full-length adapter for Illumina sequencing with further PCR amplification. PCR products were purified (AMPure XP system) and libraries were analyzed for size distribution by Agilent2100 Bioanalyzer and quantified using real-time PCR. The RNA libraries were generated using NEBNext Ultra RNA library prep kits for Illumina (cat. E7530, NEB, USA) following the manufacturer’s instructions. All the samples were sequenced based on the Illumina Hiseq PE150 platform. In total, we sequenced at least 20 Gb raw data for each DNA sample and 10 Gb raw data for each RNA sample. The reads produced by the high-throughput sequencing machine (Illumina) were evaluated and processed by FastP (v0.20.0, -c -detect_adapter_for_pe -l 75) to discard low-quality reads (Chen et al. 2018).

### Genome assembly and cleanup

DNA reads were assembled by SPAdes (v3.13.0) with “-k 21,33,55,77--careful”. “--sc” was additionally enabled for Multiple Displacement Amplification (MDA) data to deal with non-uniform coverage and to remove potential chimeras (Bankevich et al. 2012; Nurk et al. 2013; Papudeshi et al. 2017; Prjibelski et al. 2020; Xu and Zhao 2018). GapCloser (v1.1.2, a module of SOAPdenovo2) was employed to fill gaps in assemblies (Luo et al. 2012). QUAST (v4.6.3) was used to obtain N50, GC content and other genomic statistics (Gurevich et al. 2013). CD-HIT (v4.6.1, cd-hit-est -c 0.98 -r 1 -n 10) was applied for removing redundant sequences. The telomeres were identified using a custom Perl script (Code S1) that recognized the telomeric repeats at the ends of contigs (regular expression pattern of Perl: /[AC]*CCCCAA[AC]*CCAAAA/ and /GGGGTT[GT]*TTGGGG[GT]*/). Poorly supported contigs (coverage < 1) in the assemblies were also discarded. Mitochondrial genomic sequences of ciliates and the dataset of bacterial sequences were downloaded from GenBank as a BLAST database to identify contamination caused by mitochondria or bacteria (BLAST v2.10.0 + , cutoff: *E*-value <1* e*–10) (Camacho et al. 2009). For the MAC assemblies of *S*. cf. *sulcatum*, RNA-seq data were mapped to the assembly using HISAT2 (v2.1.0, --dta) (Kim et al. 2015). All contigs without telomeres or any RNA read mapping were discarded. Short (< 400 bp) contigs, except for those with both telomeres, were also removed as poorly supported sequences. For the MIC assemblies, three replicates of *H. grandinella* and six of *S*. cf. *sulcatum* were assembled separately and reassembled using CAP3 with strict overlap parameters (-o 50 -p 99) (Huang and Madan 1999) to obtain more genomic data (Lyu et al. 2023). Potential MAC contamination in the MIC assemblies, which are unavoidable during amplification, were identified using BLAST (Camacho et al. 2009) (v2.10.0 + , cutoff: *E*-value < 1*e*–10, identity > 97%, qcov > 95%) and removed by a custom Perl script (Code S2).

### Transcriptome assembly and gene prediction of the *S*. cf. *sulcatum* MAC genome

Transcripts were assembled using StringTie (v2.2.1) (Pertea et al. 2015). The *S*. cf. *sulcatum* genome assembly with all the identified introns removed was aligned against the Swiss-Prot database using BLAST (v2.10.0 + , blastx -query_gencode 6 -evalue 1*e*–5) (Camacho et al. 2009) to identify open reading frames (ORFs). The conventional stop codons TAA and TAG were modified to encode glutamine, as in other oligotrich and hypotrich ciliates (Swart et al. 2016). For the transcripts without ORFs identified, TransDecoder (v5.5.0, -G Ciliate, https://github.com/TransDecoder/TransDecoder/) was employed to extract the long ORFs and predict the likely coding regions of them. The ORFs identified by BLAST alignments were then used for building a training set for AUGUSTUS (Stanke et al. 2008), which was used to perform de novo gene prediction for the genes without assembled transcripts. Ultimately, 2078 *S*. cf. *sulcatum* genes were used to train AUGUSTUS. The transcripts assembled by StringTie were used to produce additional constraints (hints) for AUGUSTUS. For gene prediction, AUGUSTUS was run with the following parameters: “--alternatives-from-evidence = true --enemodel = complete --min_intron = 18”. Predicted protein products were annotated by alignment to domains in the Pfam-A database by InterProScan (Jones et al. 2014) and to the SwissProt database by DIAMOND (Buchfink et al. 2015) (-evalue 1*e*–5). Gene Ontology (GO) terms were enriched by using the BINGO (Maere et al. 2005) plugin (v3.0.3) in the Cytoscape platform (v3.6.0) (Shannon et al. 2003). The corrected (corr) *p*-values were derived from a hypergeometric test followed by Benjamini and Hochberg false discovery rate (FDR) correction. An FDR ≤ 0.05 was regarded as significant.

### TBE/Tec detection and estimation of substitution rates

Transposable elements were detected as described in other ciliates (Chen and Landweber 2016; Feng et al. 2022). Briefly, representative *Oxytricha* TBE ORFs (Genbank accession AAB42034.1, AAB42016.1, and AAB42018.1) were used as queries to search TBEs in the *S*. cf. *sulcatum* and *H. grandinella* MIC genomes by TBLASTN (-db_gencode 6 -evalue 1*e*–5 -max_target_seqs 100,000). Tec ORFs were similarly detected by using *E. crassus* Tec1 and Tec2 ORFs as queries (-db_gencode 10 -evalue 1*e*–5 -max_target_seqs 100,000, Genbank accessions of Tec ORFs are AAA62601.1, AAA62602.1, AAA62603.1, AAA91339.1, AAA91340.1, AAA91341.1, AAA91342.1). Regions containing three ORFs in proximity (within 2 kb of each other) and in the correct orientation were annotated as complete TBEs, while those that did not contain all three ORFs were annotated as partial TBEs. ParaAT (v2.0, -c 6 -f axt -k) (Wang et al. 2010) and KaKs_Calculator (-m GY) (Wang et al. 2010) were employed to calculate the ratios of nonsynonymous to synonymous rates (dN/dS).

### Biased nucleotide distribution

All two-telomere contigs were rearranged according to gene direction after removal of telomeric sequences. Each contig was sliced into 250 bins from the 5ʹ end to the 3ʹ end, and the frequencies of A, T, G, and C in each segment were calculated. The average frequency of the four nucleotides for each segment was counted in all chromosomes. 50 bp of the subtelomeric region at each end were extracted and a sliding window of 10 bp with a step of 2 bp was used to calculate the AT content. The same regions were used to analyze base compositions using WebLOGO3 (Crooks et al. 2004).

### Genome rearrangement analysis

The MAC assembly was aligned using BLAST against the MIC assembly to analyze the rearrangement patterns between MIC and MAC genomes after trimming telomeric repeats. The whole pipeline was similar to, but more moderate than, the MIDAS (http://knot.math.usf.edu/midas/), which was originally developed to annotate MDS/IES. The masked BLAST database was created with the MIC assembly using windowmasker (Morgulis et al. 2006). Two steps of BLAST and a custom Perl script (Code S3) were applied to generate High-Scoring Pairs (HSPs): megablast was employed first (-word_size 28 -ungapped -evalue 1*e*–5); then blastn-short (-word_size 12 -ungapped -evalue 1*e*–5) was used immediately to search smaller MDSs for any gap between HSPs after megablast. Briefly, the main steps of Code S3 were: (1) HSPs with canonical “MDS-pointer-MDS” structures were identified as MDSs; (2) MDSs which shared the same pointer were temporarily connected to longer ones; (3) all adjacent MDSs were checked, and nested ones were discarded; 4) finally, the previously merged HSPs were restored to the original status, and HSPs were identified as MDSs on both MAC and MIC. The MIC-limited sequences between two MDSs were identified as IESs. The overlaps of two adjacent MDSs on MAC sequences were denoted as pointers. Scrambled chromosomes referred to those with MDSs in different orders or orientations, and the genes encoded by them were identified as scrambled genes. Chromosome breakage sites (CBSs) were identified as the regions between the subtelomeric MDSs from different MAC chromosomes that mapped to the adjacent regions of the MIC genome. The composition of bases of the most abundant pointers and CBSs was displayed using WebLOGO3 (Crooks et al. 2004). The rearrangement patterns of chromosomes encoding the same single-copy homologous gene “Calcineurin-like phosphoesterase” in the four species were displayed using LINKVIEW (https://github.com/YangJianshun/LINKVIEW).

### Alternative processing

Two types of alternative fragmentation were identified based on the architecture of MDSs and IESs on germline contigs: (A) the same MDS region in micronuclear DNA was processed into multiple, distinct somatic chromosomes; (B) the MDS from one chromosome was nested by IES from another chromosome. MAC contigs involved in alternative fragmentation were aligned with the NCBI non-redundant protein sequence database after the removal of introns (DIAMOND-blastx -query-gencode 6 -e 1*e*–2) (Buchfink et al. 2015). The best hit of each query contig was retained.

### Identification of homologous IESs and genes

The homologous genes of *S.* cf. *sulcatum*, *H. grandinella*, *E. vannus* and *O. trifallax* were clustered using OrthoVenn2 (Xu et al. 2019) with an E-value threshold of 1*e*–5. To identify the IESs with homologous excised sites, the homologous proteins were aligned using MAFFT (v7.487, --globalpair) (Katoh and Standley 2013). The locations of pointers that locate at amino acids with the same position in alignments were identified as homologous IES-excised sites. All the IES-excised sites involved in alternative fragmentation were also removed. Another category of homologous IESs were identified based on their sequence similarities without considering their locations. All the IESs identified in the germline genomes were clustered using CD-HIT (v4.6.1, cd-hit-est -c 0.9 -s 0.9) (Fu et al. 2012) after the removal of IESs with homologous excised sites. The list of clusters outputted in a text file was processed using a custom Perl script (Code S4) to analyze the homologous IESs.

## Results

### Genome assembly and features

**Fig. 1:**
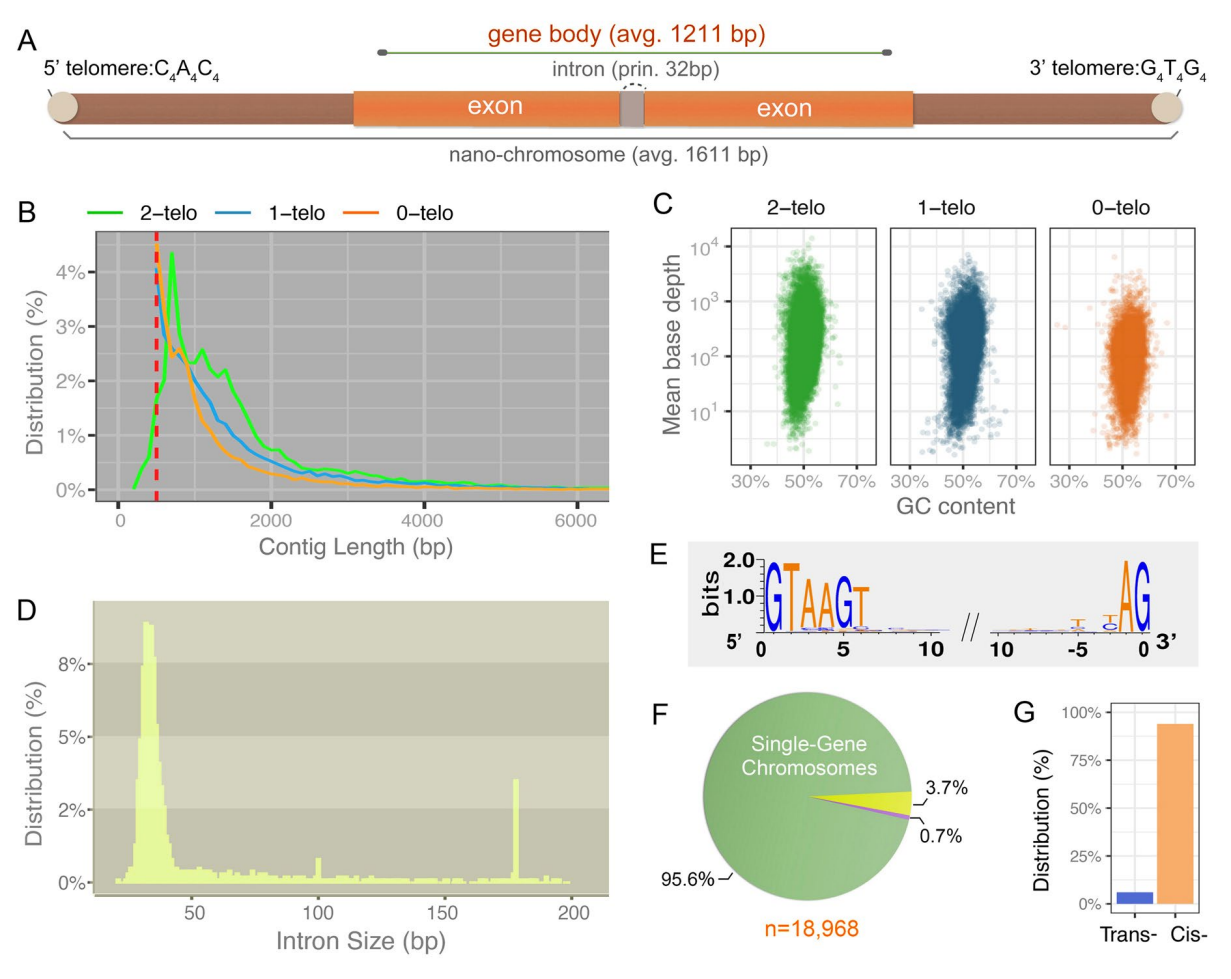
Macronucleus (MAC) genome assembly and features of chromosomes and introns of *Strombidium* cf. *sulcatum*. **A** The schema illustrates the canonical structure of nanochromosomes in the MAC of *S.* cf. *sulcatum*. **B** The size distribution of contigs with 0, 1 or 2 telomeres in *S.* cf. *sulcatum*. **C** Correlation between the GC-content (%) and mean base depth of each contig. X-axis shows the GC-content (%) and Y-axis shows the mean base depth. Each dot represents one contig. Scaffolds with either 0/1/2 telomeres share a similar GC-distribution. **D** The size distribution of 6240 identified introns. **E** The base composition of 10 nt at both ends of each intron. **F** Nanochromosomes with complete genes were assessed for gene content; only 3.7% of nanochromosomes contain two genes and 0.7% contain three or more genes. **G** The proportion of cis-nanochromosomes (two genes located on the same DNA strand) and trans-nanochromosomes (two genes located on opposite strands)

Following removal of low-quality reads, nearly identical contigs, and contaminating mitochondrial and bacterial genomes from the initial sequencing of *S.* cf. *sulcatum*, we assembled a somatic MAC genome of 71.3 Mb (Table 1; Fig. 1). The overall GC content (51.6% of all contigs and 50.7% for two-telomere contigs, Fig. 1C) is higher in this species than all other spirotrichs included in the present analysis (Table 1; Supplementary Table S1) (Chen et al. 2019; Swart et al. 2013; Zheng et al. 2021). The final assembly of the *S.* cf. *sulcatum* MAC genome contains 20,086 contigs with telomeric repeats of C_4_A_4_ and G_4_T_4_ at both ends (avg. 1611 bp, Fig. 1B), indicating a highly fragmented macronuclear genome. Complete (two-telomere) chromosomes in *S.* cf. *sulcatum* range from 257 bp to 15,209 bp. An additional 15,744 contigs contain only one telomere (avg. 1478 bp, Fig. 1B) and contigs with at least one telomere comprise 73.7% of the assembly. About half of the telomeres sequenced on the ends of each chromosome in *S.* cf. *sulcatum* are 20 bp, with a range from 12 to 141 bp (Supplementary Fig. S1A). Among all two-telomere chromosomes, ~ 78% are shorter than 2000 bp, a higher percentage than is seen in other spirotrichs with “nanochromosomes”, such as *Strombidium stylifer* (~ 54%), *Halteria grandinella* (~ 61%), *Oxytricha trifallax* (~ 35%) and *Euplotes vannus* (~ 50%).

**Table 1:** Overview of the somatic genome assemblies of *Strombidium* cf. *sulcatum* (present study), *Strombidium stylifer* (Li et al. 2021), *Halteria grandinella* (Zheng et al. 2021), *Oxytricha trifallax* (Swart et al. 2013) and *Euplotes vannus* (Chen et al. 2019)

Species	*S.* cf. *sulcatum*	*S. stylifer*	*H. grandinella*	*O. trifallax*	*E. vannus*
Assembly size [Mb]	71.3	58.8	64.0	67.2	85.1
Contigs number	48,602	29,989	39,632	22,450	38,245
N50 [bp]	1721	3073	2064	3736	2685
GC content [%]	51.62	46.34	43.11	31.36	36.89
GC content of chromosomes [%]	50.68	46.20	37.67	31.72	37.31
2-telomere contigs	20,086	19,471	16,394	15,993	25,519
1-telomere contigs	15,744	9643	12,654	5303	7835
0-telomere contigs	12,772	875	10,584	1154	4890
Telomere-containing contigs [%]	73.6	97.1	73.5	94.9	87.2
Gene number	47,569	23,580	17,815	24,885	43,338
Mean gene size [bp]	1121	940	1078	1930	749

Considering that assemblies of *Strombidium*, *Halteria* and *Euplotes* are based on short reads, the assembly of *Oxytricha* is also based on short-reads sequencing technology to make the results more comparable

The final somatic genome assembly is predicted to contain 47,569 protein-coding genes, along with 71 tRNA genes covering the standard 20 amino acids. A model for nanochromosome structure in the MAC of *S.* cf. *sulcatum* is shown in Fig. 1A. Although a small proportion of the nanochromosomes encode two (827 nanochromosomes) or more genes (109 nanochromosomes), 95.6% of *S.* cf*. sulcatum* nanochromosomes encode only one gene each (Fig. 1F). Most of the two-gene nanochromosomes of *S*. cf. *sulcatum* encode genes on the same strand (described as “cis- nanochromosomes”, Fig. 1G), which is similar to *Euplotes* (62–89%), but different from *Tetmemena* (57%), *Halteria* (49%), *Oxytricha* (41%) and *Stylonychia* (36%) (Supplementary Table S2). The single-gene nanochromosomes contain very short non-coding regions while protein-coding regions account for an average of 82% of the entire chromosome. In total, 87.7% of RNAseq reads could align with the final assembly. Potential transcription start sites (TSS) and transcription end sites (TES) of each gene were identified based on the mapping boundary of RNAseq reads. The region between the telomere and the TSS averages 48 bp and TESs are on average 47 bp from the downstream telomere (Supplementary Fig. S2A). Moreover, the introns of *S.* cf. *sulcatum* are also small in size, with a principal size distribution between 28 and 42 bp (Fig. 1D). Canonical splice site dinucleotides (5ʹ-GT…AG-3ʹ) exist at the end of these introns, with a weaker but still obvious bias of 5ʹ-AAGT-3ʹ at the 3rd to 6th nucleotides from the 5ʹ end (Fig. 1E).

Partial germline MIC genomes of *S.* cf*. sulcatum* and *H. grandinella* were acquired using single-cell whole-genome amplification techniques (Table 2). We obtained 135 Mb and 225 Mb of the MIC genome in *S.* cf*. sulcatum* and *H. grandinella*, respectively. The MIC assemblies contain 53.1% and 42.2% of the nucleotides assembled into MAC contigs in *S.* cf*. sulcatum* and *H. grandinella*, respectively. We also annotated 214 complete transposable elements (TEs) with telomere-bearing elements (TBEs) of the Tc1/mariner family, as well as 10,983 partial TBEs and 30 partial Tec elements (transposon identified in *Euplotes*) in the MIC assembly of *H. grandinella* (Supplementary Table S3). 101 partial TBEs were also found in the MIC assembly of *S.* cf*. sulcatum*. The overall dN/dS ratios of TEs are lower than 1 and mostly in the range of 0.1–0.4 (Supplementary Fig. S1E), suggesting purifying selection acting on them, which is similar to a previous study (Chen and Landweber 2016).

**Table 2:** Overview of the draft germline genome assemblies of *Strombidium* cf. *sulcatum* (present study), *Halteria grandinella* (present study), *Oxytricha trifallax* (Chen et al. 2014) and *Euplotes vannus* (Chen et al. 2019)

	*S.* cf. *sulcatum*	*H. grandinella*	*O. trifallax*	*E. vannus*
Assembly size [Mb]	135	225	496	120
Contigs number	44,899	117,978	25,720	104,988
GC content [%]	52.0	42.4	28.4	36.0
N50 [bp]	5331	2849	27,807	2566
Size (with MDS) [Mb]	65.7	80.0	216.2	78.9
N50 (with MDS) [bp]	3862	3709	45,630	3702
Identified MDS number	47,381	90,265	250,449	124,321
Mean length of MDS [bp]	927	317	273	561
Identified IES number	9786	37,159	200,357	24,674
Mean length of IES [bp]	487	368	340	406

The identification of MDS and IES of *Oxytricha* is based on its MAC assembly using short-reads sequencing technology to make the results more comparable

### Orthologous macronuclear genes in the four species of the class Spirotrichea

**Fig. 2:**
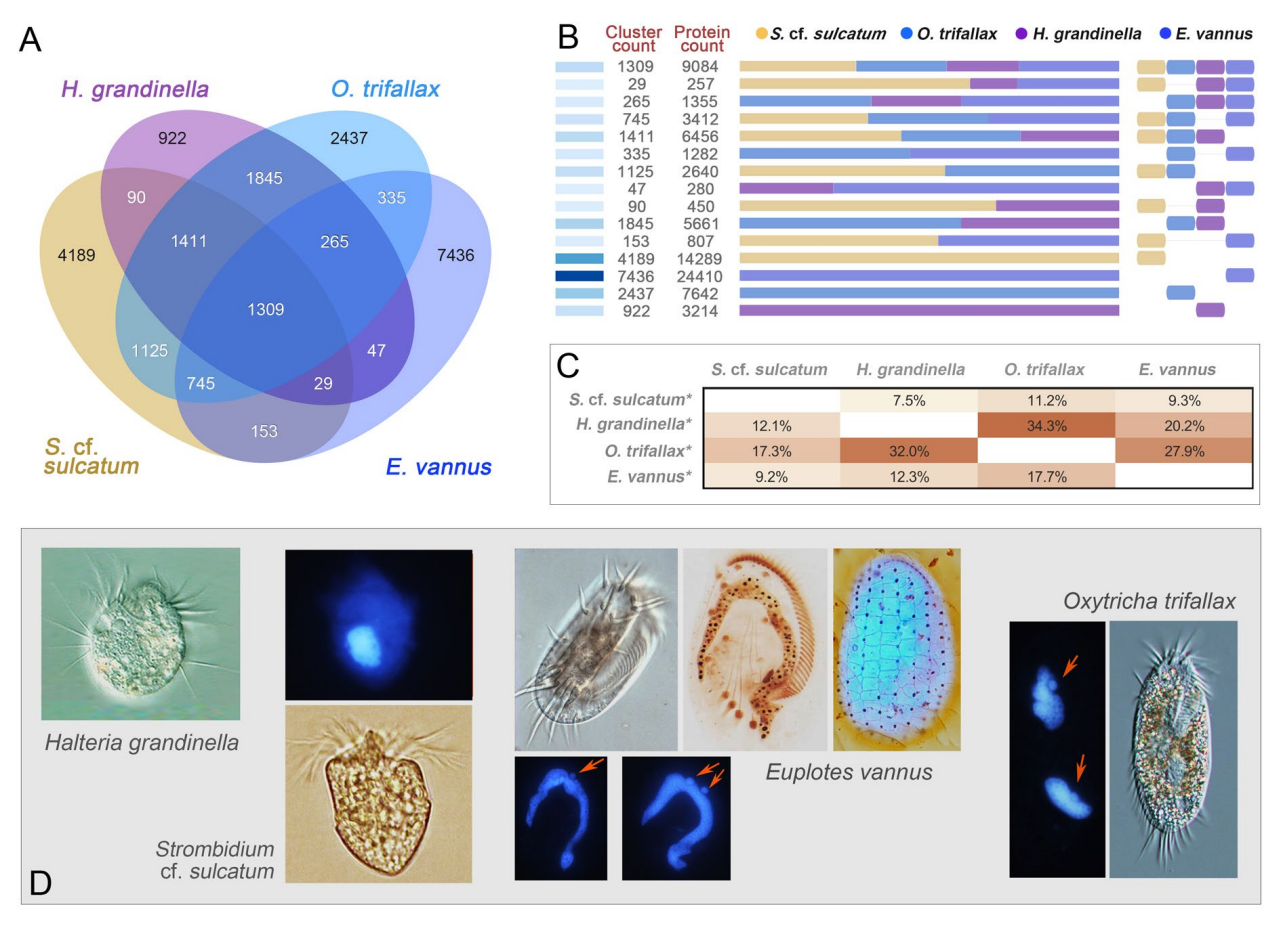
Homologous genes of *Halteria grandinella*, *Strombidium* cf. *sulcatum*, *Oxytricha trifallax* and *Euplotes vannus*. **A** Homologous clusters of the four species. The digits indicate the number of homologous clusters. **B** Number of homologous genes and clusters in each combination between the four species. Blocks on the left represent the quantity of genes, the darker the blocks the higher the number of genes. Bar graph refers to the proportion of genes from species indicated by the right blocks. **C** Pairwise comparison of genes among the four species. Each block indicates the number of homologous genes. The digits indicate the percentage of homologous genes in species with an asterisk (*) labeled. **D** Morphology of the four species involved in this study. Arrows indicate micronuclei

We analyzed the orthologous MAC genes of *H. grandinella*, *S.* cf. *sulcatum*, *O. trifallax* and *E. vannus* using OrthoVenn2 (Xu et al. 2019) to gain an insight of their genetic relationships. The four species share 1309 orthologous clusters which include 9084 genes (Fig. 2A, B). Generally, *H. grandinella*, *S.* cf. *sulcatum* and *O. trifallax* share many more orthologous genes with each other than with *E. vannus*. *Halteria grandinella* and *O. trifallax* specifically share the most orthologous groups (1845 clusters with 5661 genes), followed by that between *S.* cf. *sulcatum* and *O. trifallax* (1125 clusters with 3640 genes). Comparatively, *S.* cf*. sulcatum* shares fewer orthologous genes with *H. grandinella* (90 clusters with 450 genes) and *E. vannus* (153 clusters with 807 genes). The result of pairwise comparison of orthologous genes among the four species also shows that *H. grandinella* and *O. trifallax* share the most orthologous genes (Fig. 2C). In *H. grandinella*, more than one third (34.3%) of the genes are homologues to those in *O. trifallax*. In *S.* cf*. sulcatum*, 17.7% of the genes are homologues to those in *O. trifallax*, 12.3% to those in *H. grandinella* and 9.2% to those in *E. vannus*.

### Chromosome breakage sites are retained in the somatic genome during PGR

**Fig. 3:**
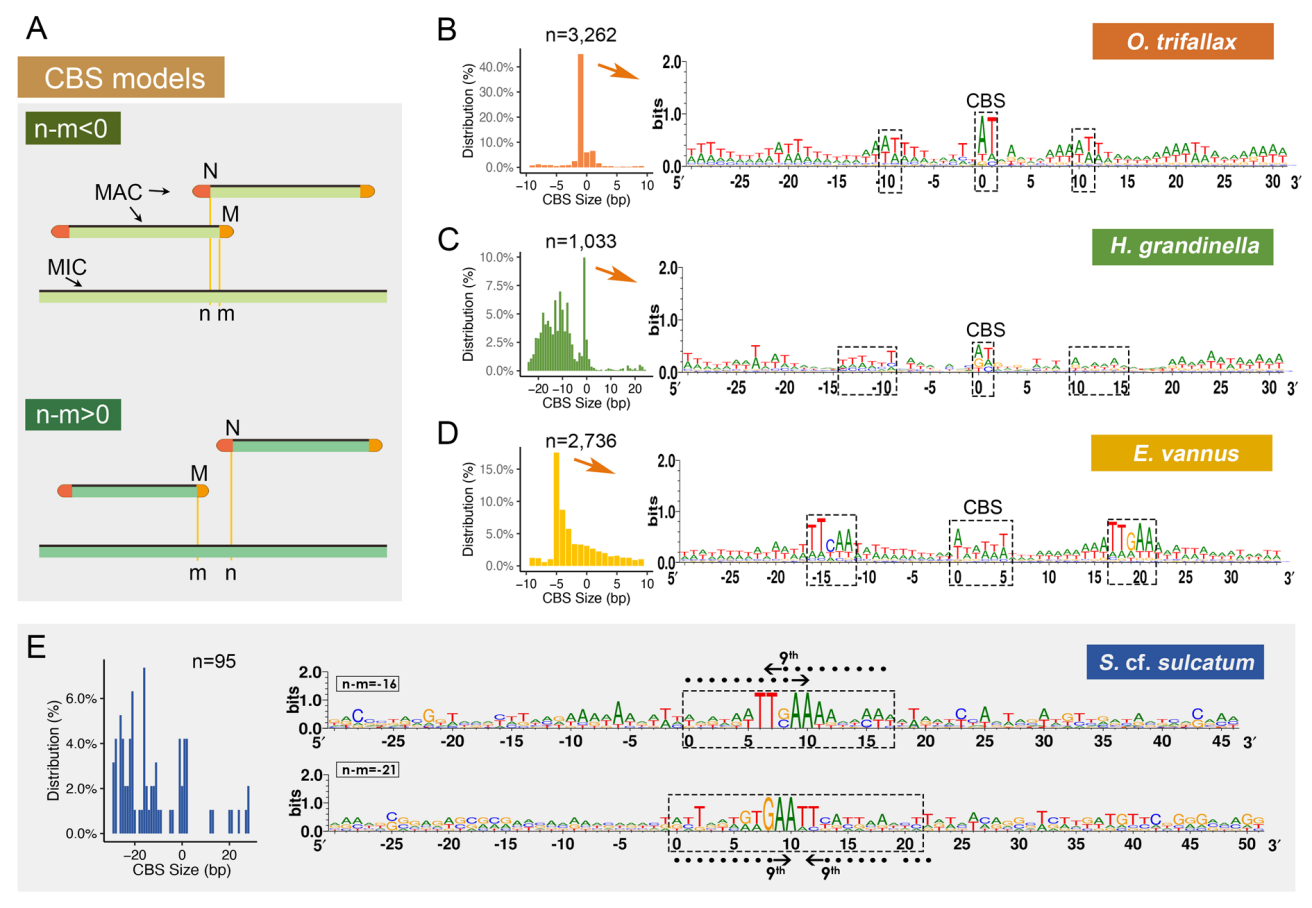
Chromosome breakage site (CBS) models of *Halteria grandinella*, *Strombidium* cf. *sulcatum*, *Oxytricha trifallax* and *Euplotes vannus*. **A** The cartoon shows models for CBS retention (top) or elimination (bottom). “m” and “n” denote the ends of two adjacent MAC chromosomes, corresponding to the breakage points “m” and “n” in the MIC genome. The CBSs are retained in somatic chromosomes if n–m < 0, while they are deleted in the case of n–m > 0. **B**–**E** The putative CBS size distribution and base composition of CBSs in the most abundant size and flanked sequences in the four species. Dashed boxes in (**B**–**E**) denote CBSs (**B**–**E**) and nearby consensus elements (**B**–**D**)

An important event that occurs during MAC development is extensive chromosome fragmentation at chromosome breakage sites (CBSs). CBSs have been found to be duplicated and retained in the MAC genome in *E. vannus* and *O. trifallax* (Chen et al. 2019). To investigate the chromosome breakage mechanism in *S.* cf. *sulcatum* and *H. grandinella*, we analyzed the subtelomeric region of their complete MAC chromosomes and the corresponding MIC sequences to identify the CBS boundaries, using the same method as described in the study of *E. vannus* (Chen et al. 2019). Briefly, the CBS region is delimited by the end of the upstream MAC chromosomes (denoted as “m”) and the beginning of the downstream MAC chromosomes (denoted as “n”) in the germline sequences. The distance between two adjacent CBS boundaries is calculated using the value of “n – m” (Fig. 3A). A positive value indicates that the CBS is not retained in the MAC genome whereas a negative value means that the CBS is retained in the MAC genome.

In total, we identified 95 and 1033 predicted CBS regions in *S.* cf. *sulcatum* and *H. grandinella*, respectively. For most CBS regions in both species, the value of “n–m” is negative, showing that the CBS region is retained in both adjacent MAC chromosomes following genome rearrangement (Fig. 3B–E). *Halteria grandinella* shares the most common motif (5ʹ-AT-3ʹ) found in CBSs of *O. trifallax*, but also possesses larger CBSs between 5 and 30 bp, which are not prominent in *O. trifallax* or *E. vannus*. However, similar to the 2 bp ‘AT’ pattern, most of these larger CBSs begin with an “A” and end with a “T”. The 30 bp flanking standard CBSs were analyzed for conserved sequence motifs using WebLogo (Crooks et al. 2004). In *H. grandinella*, these flanking regions are AT-rich, with two complementary motifs located upstream and downstream of the CBSs. This pattern is similar to those in *O. trifallax* and *E. vannus*, though the conserved motifs are different (Chen et al. 2019).

In this study we were unable to obtain as many CBSs for *S.* cf. *sulcatum* as for the other three species. However, an initial check of the regions surrounding conserved 17 bp and 22 bp motifs, for which there were more than five examples, showed reverse complement motifs at the flanking 9th to 11th bases in either direction.

**Fig. 4:**
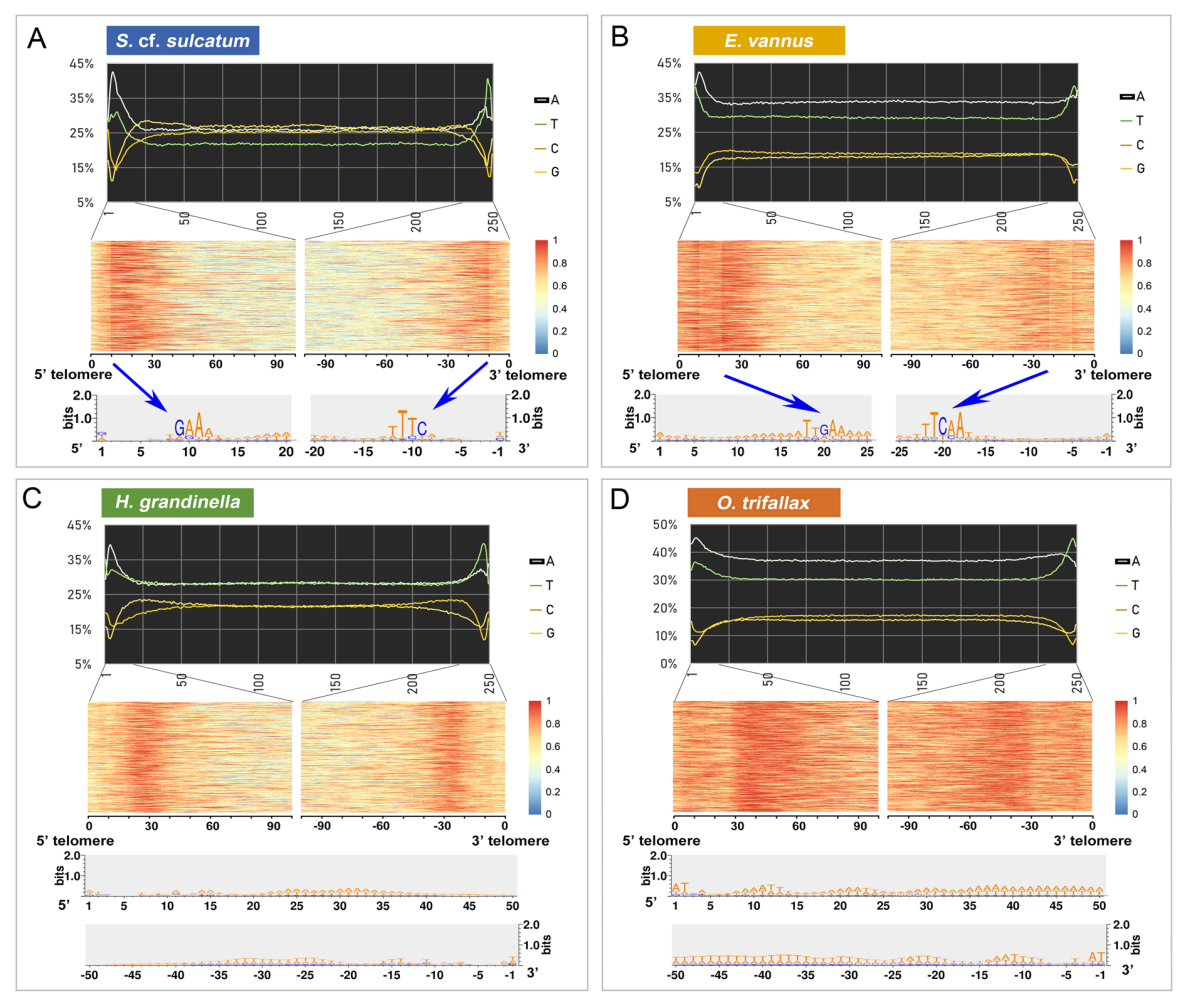
The nucleotide bias among somatic nanochromosomes and subtelomeric regions. **A**–**D** The top panels of each species show the nucleotide bias of nanochromosomes. All contigs capped with 2-telomeres were split into 250 bins after the removal of telomere sequences. The nucleotide composition of each bin was calculated and is shown as a line chart. AT-rich regions were found at both ends of the chromosomes. The terminal 100 bp of the subtelomeric regions were extracted to count the AT-content using a sliding window of 10 bp and step size of 1 bp, shown as heatmaps in the middle panels. Each row of the heatmap reflects one chromosome. The AT-content of each window is represented by the color range from warm (red) to cold (blue). Seqlogos in the bottom panels show the conserved bases of the subtelomeric region. A distinct boundary (blue arrows in **A**, **B**) in the heatmaps indicates the highly-conserved sites at the 8th to 10th nucleotides (**A**) and 18th to 22nd nucleotides (**B**) from both ends of the vast majority of nanochromosomes

To verify that the CBSs were indeed retained in the MAC genome, we focused on sequences in the subtelomeric regions of the complete nanochromosomes. The 5ʹ–3ʹ orientation of the gene found on each chromosome was determined, then the sequence was subdivided into 250 equal bins. The average content of the four nucleotides (A/T/C/G) in each bin was calculated (Fig. 4). An AT-rich bias was found in the subtelomeric regions, especially for the first and last 5% of the full length of chromosomes. We then analyzed the AT-content within the 100 bp adjacent to each telomere, revealing obvious AT hotspots at approximately 10 bp (*S.* cf. *sulcatum*), 20 bp (*E. vannus*), and 30 bp (*H. grandinella* and *O. trifallax*). These AT hotspots are independent of the gene orientation, and are present at both the 5ʹ and 3ʹ ends. The subtelomeric regions were extracted and searched for conserved sequences in each species. A highly conserved motif, 5 ʹ-GAA-3ʹ, was detected at the 9th to 11th nucleotides in *S.* cf. *sulcatum* chromosomes (Fig. 4A). A similar pattern, 5ʹ-TTGAA-3ʹ, was found in nucleotides 18–22 in *E. vannus* (Fig. 4B). By contrast, *H. grandinella* and *O. trifallax* also have an AT-rich region, but lack a conserved sequence motif at either end. These results match the sequences seen in the CBS regions in the MIC genome. Based on these observations, we conclude that all four spirotrichs share a similar retained mode of CBS breakage, with greater sequence specificity required for *H. grandinella* and *S*. cf. *sulcatum* than for *O. trifallax* and *E. vannus*.

We also considered whether the size and location of these AT-rich regions in *S.* cf. *sulcatum* may play additional roles in either transcription or translation of nanochromosome genes. Most of these regions are very short, but appear to be larger at the end containing the 5ʹ portion of the gene (avg. 17 bp for 5ʹ end vs. avg. 11 bp for 3ʹ end, Supplementary Fig. S2). The mean distance from the TSS (transcription start site) to the 5ʹ-telomere in *S.* cf. *sulcatum* is 48 bp, and 47 bp from TES (transcription end site) to 3ʹ-telomere. For single-gene chromosomes where the AT-rich regions were sequenced at both ends, more than 80% (14,391 of 17,741) of the TSSs and at least 85% of the start codons (“AUG”) on these chromosomes are located after the AT-rich region. This result was also observed in other three species (Supplementary Table S4). Since ciliates in general appear to lack a conserved TATA-box element (Brunk and Sadler 1990) and these low-complexity AT-rich regions are instead found in the chromosome where it would be expected, we speculate that these are retained in the MAC chromosomes to regulate transcription after extensive chromosome fragmentation.

### General patterns of genome rearrangements between somatic and germline genomes

The rearrangements that process the germline MIC genome during conjugation to produce the somatic MAC genome are a significant feature of ciliates (Chen et al. 2014). The patterns of rearrangement for *S.* cf. *sulcatum* and *H. grandinella* were determined and compared with those of two other spirotrichs (*E. vannus* and *O. trifallax*), whose somatic and germline genomes are both available. Considering that assemblies of both *Strombidium* and *Halteria* are based on short reads, we used the assemblies of *Euplotes* and *Oxytricha* that are also based on short-reads sequencing technology to make the results more comparable.

**Fig. 5:**
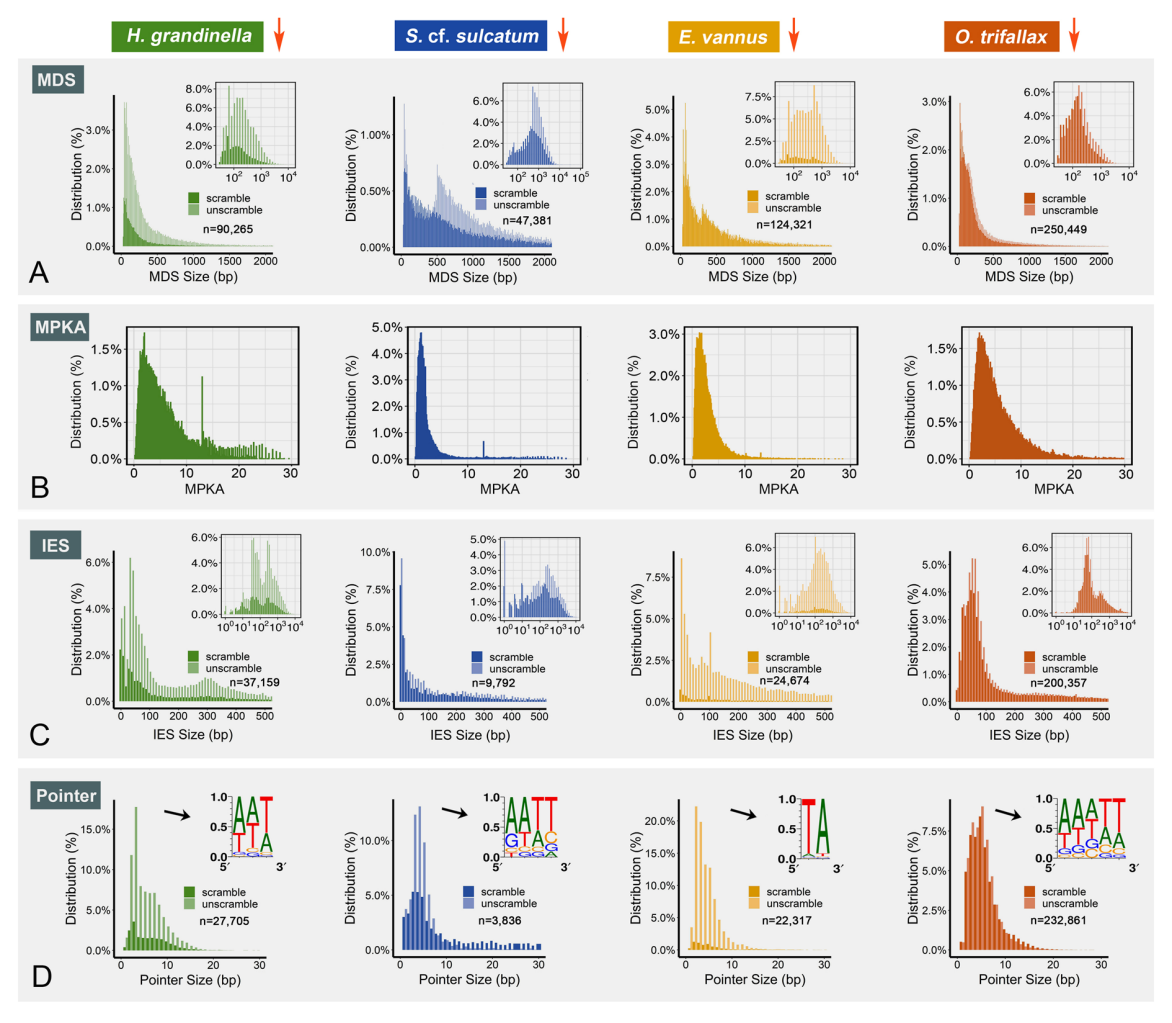
Comparison of genome rearrangements among *Halteria grandinella*, *Strombidium* cf. *sulcatum*, *Oxytricha trifallax* and *Euplotes vannus*. **A**, **C**, **D** The size distribution of scrambled and unscrambled MDSs, IESs and pointers of the four species. Data in (**A**, **C**) are displayed in two scales. The seqlogos in (**D**) refer to sequence motifs of the most abundant pointers in each species. **B** The distribution of MDS density of each MAC contig in the four species. MPKA: MDS count per kilo bases of a MAC contig

MDSs in the somatic genome assembly were identified by BLAST against the germline genome assembly. The median size of the identified MDSs is 648 bp in *S.* cf. *sulcatum*, 179 bp in *H. grandinella*, 348 bp in *E. vannus*, and 169 bp in *O. trifallax*. More than 80% of the MDSs are shorter than 500 bp in *H. grandinella* (83.5%) and *O. trifallax* (87.5%). However, that proportion is only 38.7% in *S.* cf. *sulcatum* and 64.3% in *E. vannus* (Fig. 5A). To estimate the density of MDSs, we calculated the MDS count per kilobase of a MAC contig (MPKA). The median value of MPKA is 1.45 in *S.* cf. *sulcatum*, 1.96 in *E. vannus*, 4.34 in *H. grandinella*, and 4.19 in *O. trifallax*, which indicates that the somatic nanochromosomes of *E. vannus* and *S.* cf. *sulcatum* generally consist of fewer and longer MDSs than *H. grandinella* and *O. trifallax* (Fig. 5B). This means that the somatic genomes of *S.* cf. *sulcatum* and *E. vannus* are less fragmented.

After characterizing MDSs in these species, we performed similar calculations for the IESs. The average length of an IES is shorter than an MDS, and the diversity in size among species is not as pronounced as it is for MDSs (Fig. 5C). The median IES size we identified was 180 bp in *S.* cf. *sulcatum*, 172 bp in *E. vannus*, 104 bp in *H. grandinella* and 73 bp in *O. trifallax*, though it is possible that the small size of IESs may be biased by short-reads sequencing. In order to understand the evolution of IESs in spirotrichs, we compared homologous IESs across the four species. We first identified IESs inserted at homologous sites in homologous genes among all four species (“conserved IESs”). *Halteria grandinella* (0.9%, 247 of 27,705) and *S.* cf. *sulcatum* (1.3%, 52 of 3857) contain few conserved IESs. However, both *O. trifallax* (14.9%, 34,741 of 232,861) and *E. vannus* (15.6%, 2471 of 22,317) show ten times of this number. The majority of conserved IESs in all species are found in paralogous genes within the same species (99.5% in *E. vannus*, 84.6% in *S*. cf. *sulcatum*, 73.4% in *H. grandinella* and 70.9% in *O. trifallax*). Comparing orthologous gene clusters with conserved IES across species, nearly all (77/78) are shared by *O. trifallax* and another species: *O. trifallax* and *H. grandinella* (61; 78.2%), *O. trifallax* and *S*. cf. *sulcatum* (9; 11.5%), and *O. trifallax* and *E. vannus* (7; 9.0%).

We then searched for IESs that share high sequence similarity, but are located in different (nonhomologous) loci (“mobile IESs”). There are few mobile IESs in *H. grandinella* (1.1%, 374 of 35,048), *E. vannus* (1.8%, 397 of 22,019) and *S.* cf. *sulcatum* (2.7%, 220 of 8173), but they make up a large portion of the IES population in *O. trifallax* (31.5%, 60,330 of 191,263). In total, we identified 207 mobile IESs shared among the four species, with more than a half (117, 56.5%) of these shared by *H. grandinella*, *S.* cf. *sulcatum* and *E. vannus* (Supplementary Table S5).

Pointers are short sequences repeated in the MDS-IES junctions that flank an IES in the MIC, but are present in only one copy in the MAC following IES removal. Pointers were identified based on overlaps of two adjacent MDSs on somatic sequences. Most pointers in these four species are AT-rich sequences shorter than 10 bp (Fig. 5D). Most *E. vannus* pointers show a highly conserved 5ʹ-TA-3ʹ motif that has been observed in *Paramecium* species (Steele et al. 1994). *Halteria grandinella*, *S.* cf. *sulcatum*, and *O. trifallax* pointers are all more diverse, though roughly half of the pointers in each species contain at least one 5ʹ-ANT-3ʹ motif (41.9%, 47.8%, 50.6%, respectively).

### Variable levels of gene scrambling in the germline genome

**Fig. 6:**
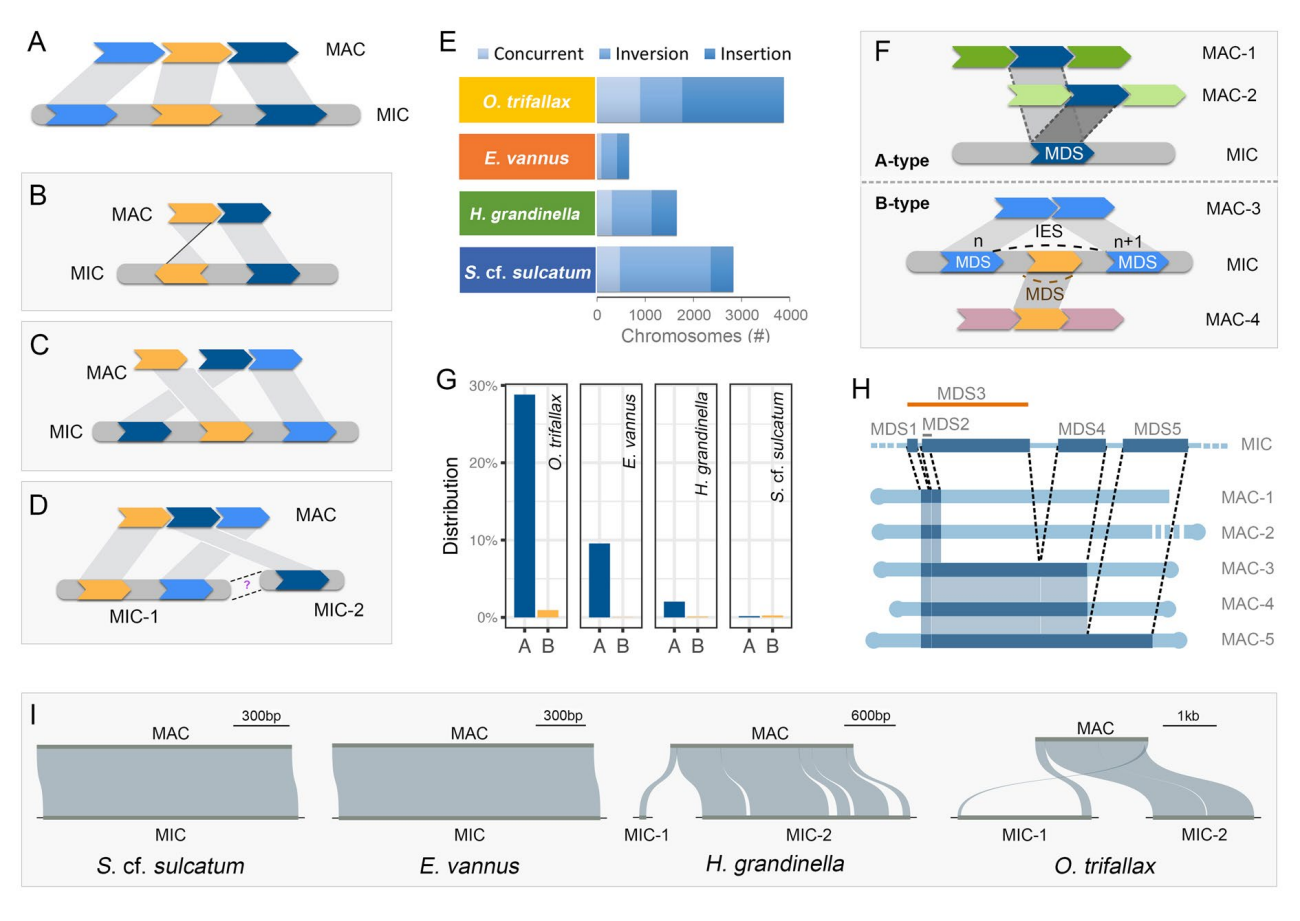
Scrambled genome rearrangement patterns in *Halteria grandinella*, *Strombidium* cf. *sulcatum*, *Oxytricha trifallax* and *Euplotes vannus*. **A** The most common pattern of unscrambled gene rearrangement. Colorful blocks indicate the MDSs, grey boxes represent the IESs. **B**–**D** Scrambled gene rearrangement models. The MDSs in (**B**) are in different orientations and the ones in (**C**) are in reverse order. The MIC-1 and MIC-2 in (**D**) represent different MIC chromosomes, or may be interrupted as a result of insufficient sequencing and is regarded as a case of (**C**) in the downstream analysis. **E** The number of scrambled chromosomes as shown in (**B**–**D**). “Concurrent” refers to chromosomes that follow either the (**B**) or **C**/**D** pattern. **F** The patterns of alternative processing events. A-type: the same MDSs in the micronuclear DNA are processed into multiple distinct somatic chromosomes. B-type: the MDS from one chromosome is nested by, or overlaps with, the IES from another chromosome. **G** The proportion of alternatively processed genes involving A-type and B-type. **H** An example case of alternative fragmentation in *Euplotes vannus*. The MDSs on one MIC contig are shared by five MAC contigs (MAC 2–5 contained both telomeres; MAC-1 had only one telomere. MDS2 is nested by MDS3). **I** The rearrangement patterns of chromosomes encoding homologs of the single-copy gene “Calcineurin-like phosphoesterase” in the four species. Other areas of the MIC are represented by short lines that are not to scale

Many MAC chromosomes (termed here “scrambled chromosomes”) in spirotrichs require a number of unscrambling events during PGR, defined as either insertion or inversion of MDSs (Fig. 6B–D). Scrambled chromosomes in the MAC genome were identified as those with one or more MDSs which are out of order in direction or position when compared with the MIC genome (Fig. 6B, C). Chromosomes with an inserted MDS from another MIC contig (Fig. 6D) are considered “insertions” (shown as Fig. 6C) in the following analysis.

All four species in this study show some degree of gene scrambling, although the levels varied widely. The highest percentage of scrambled chromosomes is in *O. trifallax* (29.2%, 5285 of 18,111), followed by *S.* cf*. sulcatum* (15.9%; 2901), *H. grandinella* (12.9%, 1693), and *E. vannus* (4.2%, 1387). The ratio of inversion to insertion events also varies among species. Scrambled nanochromosomes in *S.* cf*. sulcatum* resulting from an inversion of MDSs make up 69.4%, with 17.6% insertions, and 13.0% “concurrent” events where both occur simultaneously (Fig. 6E). Inversions are also highest in *E. vannus* (45.0%) and *H. grandinella* (37.7%), with insertions higher than concurrent events. During development, however, *O. trifallax* shows a higher rate of insertion (63.8%) than inverted (21.4%) or concurrent (14.7%).

Scrambled MDSs (13,588) accounted for 28.6% of all MDSs in *S.* cf*. sulcatum.* This is higher than the proportion in *O. trifallax* (48,689, 19.4%), which contains far more MDSs. Scrambled MDSs make up 10.6% (9569) of the MDSs in *H. grandinella* and only 4.2% (5201) in *E. vannus*. Scrambled MDSs are typically shorter than unscrambled ones. No significant difference was observed between the size of scrambled and unscrambled IESs in the four species. All four species contain more MDSs in scrambled than unscrambled nanochromosomes (*S.* cf. *sulcatum*: 2.1/kb scrambled, 1.3/kb unscrambled; *H. grandinella*: 4.9/kb scrambled, 4.2/kb unscrambled; *E. vannus*: 2.8/kb scrambled, 1.9/kb unscrambled; *O. trifallax*: 6.4/kb scrambled, 3.6/kb unscrambled).

Gene Ontology (GO) analysis of scrambled genes was performed with BiNGO (Maere et al. 2005). In *S*. cf. *sulcatum*, scrambled genes are enriched significantly for signal transduction (GO:0007165, *P*-value 6.9943* e*–7), transmembrane transporter activity (GO:0022857, *P*-value 8.9672* e*–3) and kinase activity (GO:0016301, *P*-value 1.4298 *e*–2). The scrambled genes in *E. vannus* are enriched significantly in copper ion binding (GO: 0005507, *P*-value 3.0387* e*–2) and those in *O. trifallax* are enriched in lipopolysaccharide biosynthetic process (GO: 0009103, *P*-value 1.8318* e*–2). No significant enrichments of scrambled genes were found in *H. grandinella*.

### Diverse frequency of alternative processing in Spirotrichea

To explore more diverse DNA rearrangement patterns, we identified a more complex form of DNA processing, i.e., “alternative processing”, which occurs during PGR and is analogous to RNA alternative splicing. Alternative processing was reported in *O. trifallax* (Braun et al. 2018; Chen et al. 2014, 2015; Lindblad et al. 2019) and the phyllopharyngean ciliate *Chilodonella uncinata* (Gao et al. 2014, 2015; Katz and Kovner 2010). We identified two types of alternative processing (Fig. 6F). In the “A-type”, the same MDS regions in micronuclear DNA are processed into multiple, distinct somatic chromosomes. In the “B-type”, the MDS from one chromosome is nested within the IES from another chromosome. We observed a relatively high frequency of alternative processing in *O. trifallax* and *E. vannus*, as well as some cases in *S.* cf. *sulcatum* and *H. grandinella*. Generally, the A-type cases are much more abundant than the B-type.

Among genes whose MDS structure has been defined in *O. trifallax*, 7130 involve A-type processing and 236 involve B-type (Fig. 6G). In *E. vannus,* only 4011 genes involve A-type processing and 16 involve B-type. Very few cases of alternative processing were observed in *H. grandinella* and *S.* cf. *sulcatum*. In *H. grandinella*, 269 involve A-type and 15 involve B-type. *Strombidium* cf. *sulcatum* has only 33 A-type processing events and 54 B-type. These numbers could be underestimated because we used a very strict pipeline to identify the IESs and only non-scrambled IESs are considered here. Furthermore, short contigs may prevent identification of some cases.

About 70%-90% alternative processing events disrupt the coding regions and about 75%-95% of the alternatively processed nanochromosomes are transcribed (Supplementary Fig. S3). The number of transcribed genes could be underestimated, considering that only vegetative cells were used for RNA-seq while some genes could have developmental or stress-induced expression. One of the most diverse cases we observed is shown in Fig. 6H, where one MIC contig is processed into five MAC contigs in *E. vannus*. Four of the five contigs sequenced have two telomeres, while the other has only one. Sequence similarity among them is low except for the regions from the same MIC contig. Of the five MAC contigs, four have only one detected coding region, which is predicted to encode a G1/S-specific cyclin-E protein. The other contains the G1/S-specific cyclin-E gene along with a gene predicted to encode a SWI/SNF complex component snf12 homolog. Cyclin and snf12 homologs are typically involved in the cell cycle. We also found that the MAC contigs alternatively processed from the same MIC contigs generally belong to the same gene families (80.6% in *E. vannus*, 92.4% in *O. trifallax*, 73.9% in *H. grandinella* and 68.4% in *S.* cf. *sulcatum*), probably due to the repeated usage of the same gene regions.

## Discussion

In this study, we assembled a high-quality MAC genome of the spirotrich ciliate *Strombidium* cf. *sulcatum* (which contains “gene-sized” nanochromosomes) and partial germline genomes of *S.* cf. *sulcatum* and *Halteria grandinella*. We characterized the PGR features of *S*. cf. *sulcatum* and *H. grandinella* by comparing their somatic genome to their germline genome. These features were compared with the two sequenced ciliates in the same class, *Euplotes vannus* and *Oxytricha trifallax*, to provide a holistic and evolutionary view of MIC and MAC structures in spirotrichs and to gain an insight of the diversity and architecture of PGR in ciliates. As a result, we demonstrated that: (1) somatic genomes comprised of “gene-sized” nanochromosomes are a common feature in a wide range of spirotrichs and those in oligotrichs are the most compact; (2) micronuclear chromosomes in spirotrichs are highly fragmented during macronuclear development at chromosome breakage sites which are duplicated and retained in the somatic genome; (3) the micronuclear-limited DNA may have originated as remnants of transposable elements or as degenerated duplicate sequences after gene duplication, some of which may have occurred prior to the last common ancestor of spirotrichs; (4) gene scrambling and alternative processing are widespread in spirotrichs and may play important roles in increasing genetic diversity.

### Highly fragmented and compact macronuclear genomes are a common feature of spirotrichs

Based on the available somatic genome data, the extent of chromosomal fragmentation varies dramatically among ciliates, from limited, e.g., the well-studied genera in the class Oligohymenophorea, *Tetrahymena* (Sheng et al. 2020) and *Paramecium* (Aury et al. 2006), to extensively, e.g., species in the class Spirotrichea, *Oxytricha* (Swart et al. 2013), *Euplotes* (Chen et al. 2019) and *Strombidium* (present study). Gene-sized chromosomes have been reported in disparate classes other than Spirotrichea, e.g., *Chilodonella uncinata* in the class Phyllopharyngea and *Metopus palaeformis* and *Nyctotherus ovalis* in the class Armophorea (Riley and Katz 2001), indicating that gene-sized chromosomes originated multiple times within ciliates. Of the seven subclasses in Spirotrichea, somatic genome data have been reported in three subclasses, including *Oxytricha*, *Tetmemena*, *Urostyla*, *Paraurostyla*, *Laurentiella*, *Sterkiella* and *Stylonychia* in the subclass Hypotrichia (Aeschlimann et al. 2014; Chen et al. 2015; Feng et al. 2022; Swart et al. 2013), *Euplotes* in the subclass Euplotia (Chen et al. 2019, 2021; Feng et al. 2022; Jin et al. 2023; Mozzicafreddo et al. 2021; Vinogradov et al. 2012; Wang et al. 2016), and *Strombidium* in the subclass Oligotrichia (Li et al. 2021 and present study), as well as in *Halteria*, an extremely specialized “oligotrich‐like” hypotrich (Wang et al. 2019; Zheng et al. 2021) (Supplementary Table S1). Each of these somatic genomes is about 50 to 110 Mb in size and is composed of gene-sized chromosomes that are highly enriched with coding sequences.

In the present study, we assembled the 71.3 Mb somatic MAC genome of *S*. cf. *sulcatum* comprising more than 20,000 gene-sized nanochromosomes, of which nearly 16,000 contigs contain at least one telomere. Compared with other reported ciliates with extensively fragmented somatic genomes, *S*. cf. *sulcatum* has significantly shorter nanochromosomes, with an N50 of only 1721 bp (Supplementary Fig. S1B; Table 1). Conversely, the average gene size in *S*. cf. *sulcatum* (1121 bp) is larger than in most species, except in *O. trifallax* (1930 bp, Supplementary Fig. S1C). The untranscribed region before the transcription start site (TSS) averages 48 bp, with 47 bp found after the transcription end site (TES) on single-gene chromosomes (Supplementary Fig. S2A). This means that there are fewer “extraneous” nucleotides at the ends of nanochromosomes in this species. Moreover, the majority of genes (~ 86%) in *S*. cf. *sulcatum* are intron-free, and each gene contains only 0.16 introns on average, which is less than that of *H. grandinella* (0.34), *Tetmemena* (1.09), *O. trifallax* (1.74), *E. woodruffi* (2.23) and *E. vannus* (4.05) (Chen et al. 2019; Feng et al. 2022; Swart et al. 2013; Zheng et al. 2021). This makes the macronuclear genome of *S*. cf. *sulcatum* the most compact and efficient described to date in spirotrichs. Considering that both *S*. cf. *sulcatum* and *H. grandinella* are typically planktonic, these observations highlight the apparent importance of genome compaction for growth in planktonic ciliates, although the evolutionary pressures that have led to these extremes are not known.

The coding sequence-rich nature of *S*. cf. *sulcatum* helps to explain the high (over 50%) GC content of its macronuclear genome, which is higher than that of other reported ciliates (i.e., 20–40%, Supplementary Table S1). Except for the telomeric and subtelomeric regions, where the AT content increases sharply, the GC composition is relatively uniform. Previous research about codon usage in *Strombidium sulcatum* has also shown a bias toward more GC-rich codons than others that encode the same amino acid, which may be driven by its high GC composition (Knight et al. 2001; Wang et al. 2019). In addition, the GC contents of both *H. grandinella* and *S*. cf. *sulcatum* MIC assemblies are also higher than those of *O. trifallax* and *E. vannus* (Table 2). This suggests that both MAC and MIC genomes of *H. grandinella* and *S*. cf. *sulcatum* feature high GC composition, which may be also related to their typical planktonic life style.

### The highly fragmented macronuclear genome is generated by developmental fragmentation of the micronuclear chromosomes at chromosome breakage sites

The levels of chromosome breakage during the development of somatic genomes vary dramatically among ciliates, and result in different levels of chromosomal fragmentation in the MAC genome. For spirotrichs, whose MAC genomes are composed of gene-sized chromosomes, chromosome breakage has to occur much more frequently than those with more limited fragmentation. The chromosome breakage mechanisms also differ among ciliates. In the well-studied model organism *Tetrahymena*, chromosomal breakage occurs at a conserved 15 bp sequence designated as the chromosome breakage sequence (CBS), which is necessary and sufficient to specify a breakage site (Yao et al. 1987, 1990). The CBSs and some flanking micronuclear DNA is eliminated during this process. Similarly, chromosome breakage in another model ciliate (*Paramecium*) occurs every several hundred kilobases. However, no conserved CBS has been identified and the breakage is considerably less precise, resulting in variable fragmentation sites and heterogeneity of the resulting MAC chromosomes (Baroin et al. 1987; Caron 1992). By comparison, previous findings focusing on *E. vannus* suggest that CBSs are duplicated and retained in the somatic genome (Baird and Klobutcher 1989; Chen et al. 2019; Klobutcher et al. 1998).

In addition to the consensus CBS motifs, there is a highly conserved motif flanking both sides of the CBS in an overall palindrome structure. Similar models are revealed in *H. grandinella* and *O. trifallax* in the present study, although the consensus CBS and the flanking conserved motifs are slightly different (Fig. 3). No obvious CBS and flanking conserved motif were identified for *S.* cf. *sulcatum*, although this may be due to the fact that we were unable to obtain as many CBSs as we did for the other three species. However, for the CBSs we did manage to identify, most are retained in the MAC genome. For all the studied spirotrichs in the present work, the CBS and its flanking conserved motif are also detected in the subtelomeric regions in their somatic nanochromosomes (Fig. 3). Although no obvious CBS was found in *S.* cf. *sulcatum*, a 5ʹ-GAA-3ʹ motif in the 9th–11th nucleotides at the subtelomeric regions of the MAC nanochromosomes is very obvious, and shares the same sequence with the two most abundant CBSs found in this species (Figs. 3E, 4). As the CBSs and their flanking regions are generally AT-rich (Cavalcanti et al. 2004), they might serve as potential TATA-boxes to initiate transcription (Zheng et al. 2018), with heterogeneity in these sequences allowing for the coregulation of specific genes.

### The compact macronuclear genome results from extensive removal of micronuclear-limited DNA

In addition to chromosome fragmentation, IES excision is another important event during genome rearrangements from germline to somatic genomes. Previous studies indicate that ~ 30% of the germline genome of *Tetrahymena* is eliminated during genome rearrangements (Hamilton et al. 2016), whereas ~ 90% of the germline genomes in spirotrichs is eliminated (Chen et al. 2014; Prescott 1994). The number and size of MDSs/IESs also show great variability among ciliates. Approximately 12,000 IESs, ranging from 136 bp to 43.4 kb with a median length of 2.8 kb, were predicted in *Tetrahymena* (Hamilton et al. 2016). Comparatively, the number of IESs in the spirotrich *O. trifallax* (> 225,000) is much higher than that in *Tetrahymena*, but the length is much smaller, with a median length of about 70 bp (Chen et al. 2014). Similarly, *E. vannus*, *S.* cf. *sulcatum* and *H. grandinella* also feature smaller IESs, some of which are even less than 20 bp (Fig. 5), though it is possible that the small size of IESs may be biased by short-reads sequencing. Moreover, the IESs in spirotrichs are mostly located in the protein-coding regions that require precise elimination. This contrasts with IESs in *Tetrahymena,* most of which reside in non-coding regions and are imprecisely excised from the genome, although a small number of scrambled IESs were reported recently (Sheng et al. 2020),

The high number of IESs dispersed throughout the germline genome fragments it into a large number of MDSs. Comparatively, the nanochromosomes in *H. grandinella* and *O. trifallax* contain more but shorter MDSs (about four MDSs per kb) than are seen in *E. vannus* and *S.* cf. *sulcatum* (1.5–2.0 MDSs per kb)*.* One example that highlights these differences is the recombination patterns of the single-copy homologous chromosome encoding “Calcineurin-like phosphoesterase” in the four species. The MAC nanochromosomes containing this gene in both *S*. cf. *sulcatum* and *E. vannus* consist of only one MDS, while the nanochromosomes in *H. grandinella* and *O. trifallax* are separated ​​into six and four MDSs, respectively (Fig. 6I).

The high number of IESs in spirotrichs makes the origin and evolution of IESs complex. It was previously proposed that IESs might be degenerated remnants of transposable elements (TEs) (Klobutcher and Herrick 1995, 1997), or that they could have originated from degraded duplicate sequences following gene duplication events (Ehrenfeucht et al. 2007; Feng et al. 2022; Gao et al. 2015). The 8 bp consensus 5ʹ-TAYAGYNR-3ʹ on the boundary of IESs in *Paramecium* (Sellis et al. 2021) and domesticated transposase genes from the piggyBac family in *Paramecium* and *Tetrahymena* lineages (Baudry et al. 2009; Cheng et al. 2010) support the proposal of Klobutcher and Herrick (1995) that many IESs could have resulted from transposons via “IBAF” (invasion, bloom, abdicate, and fade). The identification of several families of mobile IESs that generated tens to thousands of new copies in the *Paramecium* germline genome supports the hypothesis that TEs account for the massive proliferation of IESs (Sellis et al. 2021). In *O. trifallax*, the discovery that some transposase genes are expressed during PGR, combined with the observation that the silencing of these genes leads to abnormal DNA rearrangement, also indicates a transposon-related origin for PGR in ciliates (Nowacki et al. 2009). In the present study, we detected TEs (Supplementary Table S3) and “mobile IESs” (Supplementary Table S5) in spirotrichs, especially in *O. trifallax*, which further supports the origin of IESs from transposons. We also identified mobile IESs shared among the four spirotrichs. Considering that IESs generally evolve rapidly, and that these shared mobile IESs have a high level of sequence similarity, they must have resulted from the recent invasion of mobile elements, which may be acquired via horizontal transfer as revealed in *Paramecium* (Sellis et al. 2021). In addition to mobile IESs, we detected some other IESs inserted at homologous sites in protein-coding sequences in spirotrichs, which was also reported by Feng et al. (2022). This indicates that these shared IESs may have arisen in the germline genome of the last common ancestor of spirotrichs. However, the number of shared IESs among all spirotrichs are very limited, while hypotrichs share more conserved IESs, e.g., *Oxytricha* and *Halteria* (present study), or *Oxytricha* and *Tetmemena* which both contain a high copy number of telomere-bearing element transposons and share conserved DNA rearrangement junctions (Feng et al. 2022). These findings further support the origin of IESs from transposons and indicate that the ancestor of hypotrichs gained some IESs after its divergence from the other spirotrichs. Furthermore, many IESs are found at homologous sites within paralogous genes of a given species, which indicates that these IESs were gained after speciation but before gene duplication.

It is noteworthy that the vast majority of IESs in spirotrichs are single copy and much smaller in size than those in oligohymenophoreans (Chen et al. 2019). One explanation for this may be the rapid evolution rate of IESs. Long IESs introduced as transposons, for example, may degenerate into much smaller, unrecognizable sequences over a short period of time. Another explanation is that different types of IESs may have different origins. The IESs in spirotrichs are mostly located in the protein-coding regions, whereas TEs are generally not inserted here due to the pressure of counterselection. According to the model proposed by Gao et al. (2015) and Chen et al. (2015), gene scrambling begins with gene duplication in the germline genome, followed by partial and reciprocal degradation of the resulting duplicate sequences over time. In this scenario, alternative processing chooses different pieces from the duplicated sequences. Parts of the gene copies that are not used will become IESs and eventually be eliminated from the somatic genome. A high frequency of alternative processing and gene scrambling events during PGR in spirotrichs and other groups has been detected in the present and previous studies (Ardell et al. 2003; Chen et al. 2014, 2019; Gao et al. 2014; Smith et al. 2020), which again support the above model and imply that the IESs in the protein-coding regions are more likely from the degradation of the duplicate sequences.

### Gene scrambling and alternative processing are extensive in the core spirotrichs

Gene scrambling has been recognized in multiple ciliate lineages, such as Spirotrichea (Ardell et al. 2003; Chen et al. 2019; Smith et al. 2020), Heterotrichea (Maurer-Alcalá et al. 2018b), Karyorelictea (Maurer-Alcalá et al. 2018b) and Phyllopharyngea (Gao et al. 2015; Maurer-Alcalá et al. 2018a). The degree of scrambling varies greatly among species, and to date large-scale scrambling has only been observed in species that also show extensive fragmentation of their somatic genome. Among species where scrambled genes have been identified previously, ~ 31% of the putative genes identified in *Chilodonella uncinata* were scrambled (Maurer-Alcalá et al. 2018a), and scrambled genes account for 15.6% of all genes in *O. trifallax*, 13.6% in *Tetmemena* sp., and 7.3% in *E. woodruffi* (Feng et al. 2022). The proportion of scrambled genes has also been estimated in *E. vannus* (4.2%), *S.* cf. *sulcatum* (15.9%) and *H. grandinella* (12.9%) in the present study. These proportions are not as high as in *O. trifallax*, *Tetmemena* sp. and *C. uncinata*, which may be due to the fact that their germline genomes are still largely unsequenced. Consequently, the distal scrambled MDSs might not have been recovered or were assembled into different contigs and considered as “nonscrambled”.

Alternative processing is another complex form of chromosome rearrangement that has been proposed as an intermediate stage in the evolution of gene scrambling (Gao et al. 2015). It was previously revealed that alternative processing is extensive among gene families within *C. uncinata* (Gao et al. 2014). It has also been reported that alternative processing of MDSs usually occurs together with scrambling (Braun et al. 2018) and produces multiple genes and chromosomes in *O. trifallax* (Chen et al. 2014). In the present study, we revealed that nearly one third (28.8%) of the non-scrambled genes with MDS annotated in *O. trifallax* and 9.6% in *E. vannus* are produced by alternative processing. Only a small portion of genes were alternatively processed in *S.* cf. *sulcatum* and *H*. *grandinella*, which might be due to the low quality of their germline genomes. Most of the alternative processing events observed are ‘‘MDS shuffling’’, while some reuse the IES of one chromosome as the MDS in another. Moreover, most alternative processing events disrupt the coding regions of the genes, the majority of which are transcribed (Supplementary Fig. S3).

Both gene scrambling and alternative processing are novel processes that enable ciliates to efficiently explore protein space by constructing modular proteins, similar to exon shuffling in other eukaryotes (Patthy 1996). Scrambled nanochromosomes are produced by more and shorter MDSs than unscrambled nanochromosomes. The disparity of MDS density is most obvious in *S.* cf*. sulcatum* (2.1 per kb for scrambled vs. 1.3 per kb for unscrambled), which indicates that scrambled genes are fragmented more frequently, and IES gains may occur during this process. The higher MDS density in turn provides more opportunity for MDS shuffling to create more proteins. Considering that chromosomes which result from alternative processing tend to encode homologous proteins of the same family, these proteins may play roles at different life stages or in various biochemical processes. Therefore, alternative processing may also play important roles in regulating expression of genes on gene-sized chromosomes.

### Supplementary Information

Below is the link to the electronic supplementary material.

Supplementary file1 (DOCX 951 KB)

Supplementary file2 (XLSX 3882 KB)

Supplementary file3 (XLSX 25 KB)

## Data Availability

The Illumina sequencing data and assemblies were deposited in GenBank (Bioproject PRJNA1035826). All custom scripts used in the current study have been deposited in GitHub repository (https://github.com/Liping-L/Spirotrichs-PGR).
