# Supplementary material for: From germline genome to highly fragmented somatic genome: genome-wide DNA rearrangement during the sexual process in ciliated protists
Source: Mar Life Sci Technol. 2024 Feb 12;6(1):31–49. doi: 10.1007/s42995-023-00213-x (PMC10901763; doi:10.1007/s42995-023-00213-x)
Supplement: Supplementary file 1 — Supplementary file1 (DOCX 951 KB) [file 42995_2023_213_MOESM1_ESM.docx]

Marine Life Science & Technology

Research Paper

Running Title: Genome-wide DNA rearrangement in ciliates

**From germline genome to highly fragmented somatic genome: genome-wide DNA rearrangement during the sexual process in ciliated protists**

Liping Lyu^1^, Xue Zhang^1^, Yunyi Gao^1^, Tengteng Zhang^1^, Jinyu Fu^1^, Naomi A. Stover^2^, Feng Gao^1,3,*^

^1^ Key Laboratory of Evolution & Marine Biodiversity (Ministry of Education), and Institute of Evolution & Marine Biodiversity, Ocean University of China, Qingdao 266003, China

^2^ Department of Biology, Bradley University, Peoria, IL 61625, USA

^3^ Laoshan Laboratory, Qingdao 266237, China

*Corresponding author: Email: [gaof@ouc.edu.cn](mailto:gaof@ouc.edu.cn); ORCID: 0000-0001-9395-0125


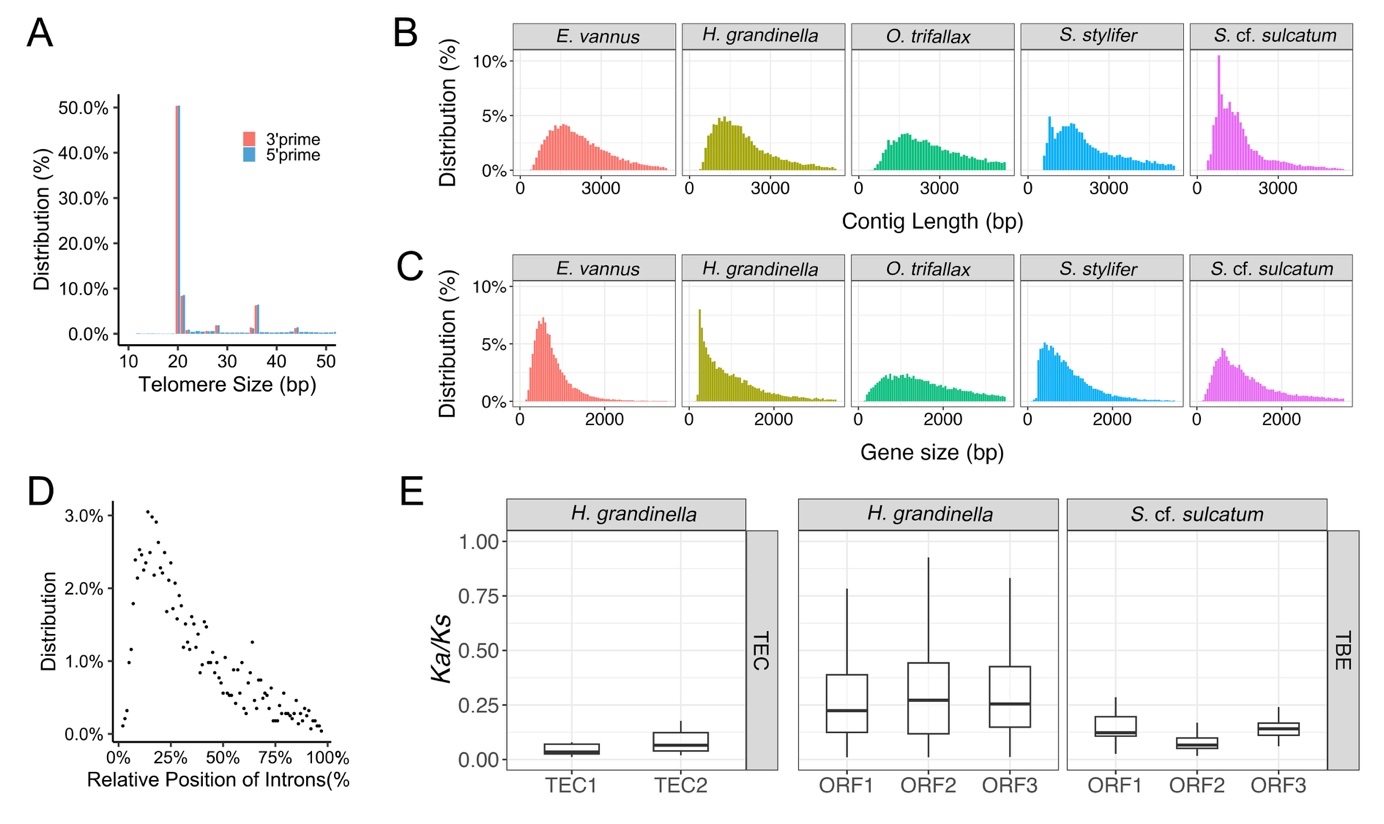


Fig. S1 (**A**) Distribution of telomere size in *Strombidium* cf. *sulcatum*. (**B**) Size distribution of somatic chromosomes in *Euplotes vannus* (avg. 2375 bp, med. 1991 bp), *Halteria grandinella* (avg. 2102 bp, med. 1722 bp), *Oxytricha trifallax* (avg. 3185 bp, med. 2572 bp), *Strombidium* *stylifer* (avg. 2381 bp, med. 1887 bp) and *Strombidium* cf. *sulcatum* (avg. 1612 bp, med. 1264 bp). (**C**) Size distribution of genes in *E. vannus* (avg. 749 bp, med. 621 bp), *H. grandinella* (avg. 1078 bp, med. 777 bp), *O. trifallax* (avg. 1930 bp, med. 1482 bp), *S.* *stylifer* (avg. 940 bp, med. 759 bp) and *S.* cf. *sulcatum* (avg. 1327 bp, med. 972 bp). (**D**) The relative position skew of introns in *S*. cf. *sulcatum*. The X-axis indicates the relative position on the coding strand of nanochromosomes. (**E**) The estimation of substitution rates (dN/dS) of transposable elements.


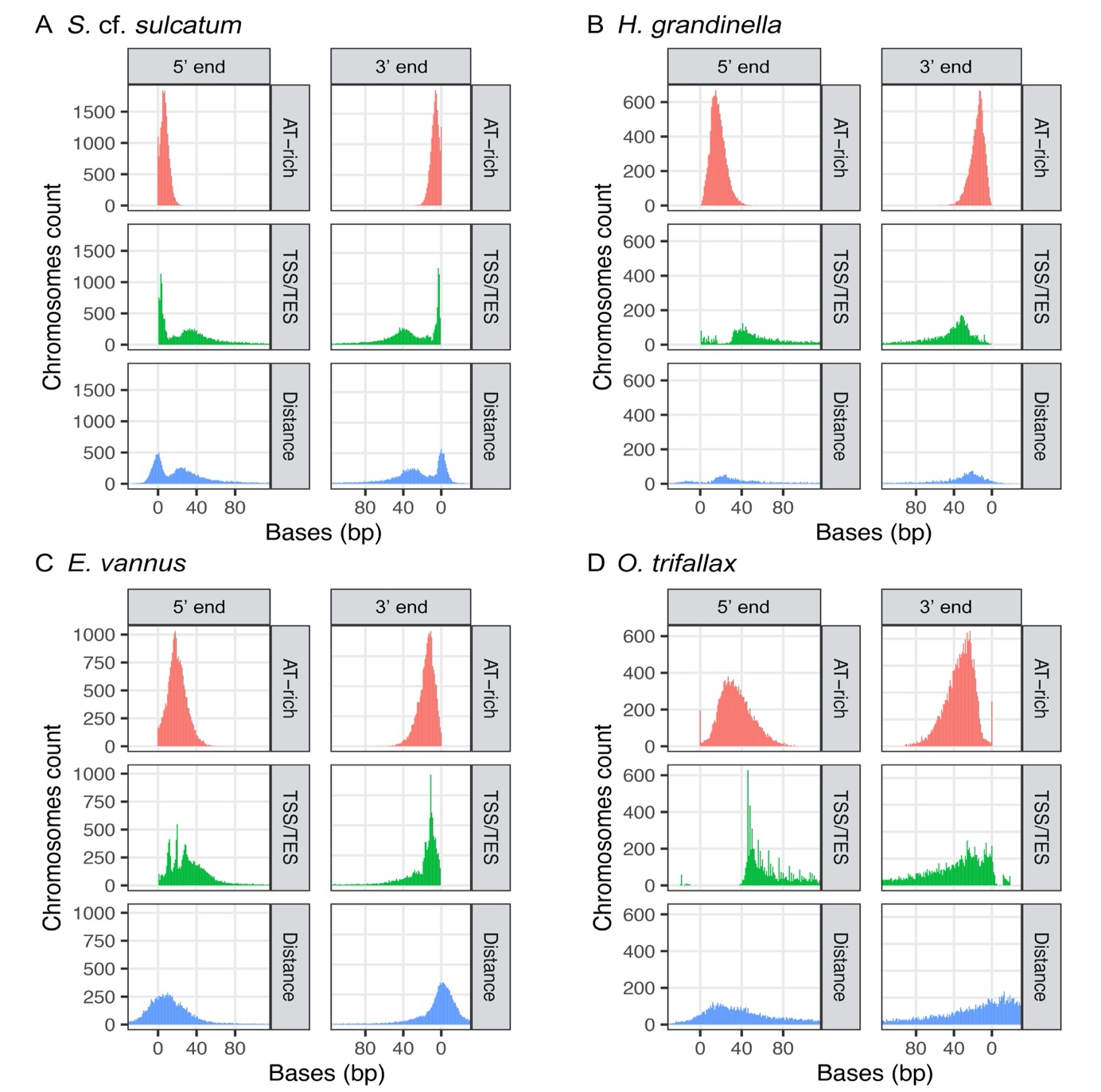


Fig. S2 Distribution of the size of AT-rich subtelomeric regions in somatic chromosomes (first row), position of TSSs (transcription start sites)/TESs (transcription end sites) (second row) and distance between TSS/TES and AT-rich region (third row) in *Strombidium* cf. *sulcatum* (**A**), *Halteria grandinella* (**B**), *Euplotes vannus* (**C**) and *Oxytricha trifallax* (**D**). “0” on the X-axis indicates the boundary of the telomere. Distance in the third row is calculated by subtracting the size of AT-rich regions from the length between TSS/TES and telomeres. Positive values indicate that TSS/TES are located after AT-rich regions.


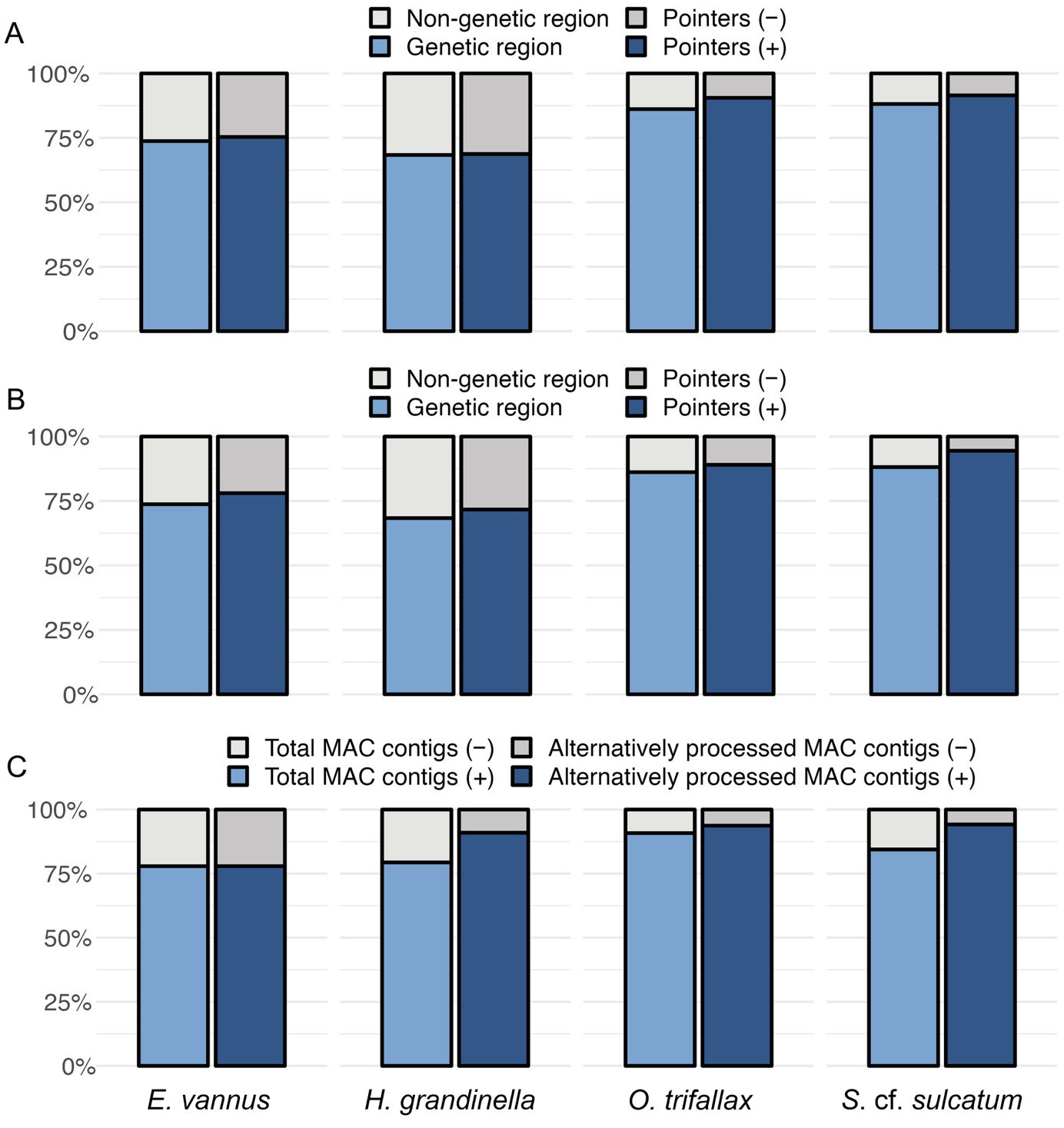


Fig. S3 The proportion of gene regions and non-gene regions on alternatively processed (**A**) MAC chromosomes and the distribution of pointers located at gene region (Pointers (+)) or not (Pointers (−)). (**B**) The proportion of MAC contigs for which the transcripts were detected (+) or not (−).

| Species | Bases / Mb | Contigs | GC | N50 / bp | Mean / bp | 5′ Telomere | 3′ Telomere | Both Telomeres |
| --- | --- | --- | --- | --- | --- | --- | --- | --- |
| *Certesia quadrinucleata* ^e^ | 71.08 | 60,709 | 42.01% | 1290 | 1171 | 605 | 940 | 17 |
| *Diophrys* sp. ^e^ | 91.37 | 39,294 | 35.31% | 2617 | 2325 | 368 | 373 | 8 |
| *Euplotes* cf. *woodruffi* ^e^ | 81.45 | 45,610 | 35.51% | 2796 | 1786 | 616 | 610 | 22 |
| *Euplotes octocarinatus* ^g^ | 88.90 | 41,980 | 28.14% | 2947 | 2118 | 31,616 | 31,869 | 29,107 |
| *Euplotes parawoodruffi* ^e^ | 74.21 | 53,384 | 39.10% | 1777 | 1390 | 573 | 508 | 15 |
| *Euplotes vannus* ^a^ | 85.09 | 38,245 | 36.86% | 2685 | 2225 | 28,627 | 29,838 | 25,058 |
| *Euplotes weissei* ^e^ | 82.14 | 86,640 | 40.31% | 942 | 948 | 4984 | 5326 | 944 |
| *Euplotes woodruffi* ^b^ | 72.17 | 35,099 | 36.55% | 2686 | 2056 | 23,085 | 24,430 | 19,148 |
| *Halteria.grandinella* ^c^ | 64.05 | 40,422 | 43.08% | 2066 | 1585 | 23,095 | 23,151 | 16,507 |
| *Laurentiella* sp. ^f^ | 49.04 | 21,383 | 28.75% | 3043 | 2293 | 17,789 | 17,766 | 16,399 |
| *Oxytricha trifallax* ^d^ | 67.17 | 22,450 | 31.35% | 3735 | 2992 | 18,254 | 18,345 | 15,531 |
| *Paraurostyla* sp. ^f^ | 57.1 | 25,391 | 29.77% | 2882 | 2249 | 21,028 | 21,019 | 19,135 |
| *Sterkiella histriomuscorum* ^f^ | 66.36 | 32,996 | 28.42% | 2822 | 2011 | 19,368 | 20,756 | 16,924 |
| *Strombidium* cf. *sulcutum* ^i^ | 72.88 | 48,647 | 51.61% | 1758 | 1498 | 27,658 | 28,267 | 20,087 |
| *Strombidium stylifer* ^h^ | 70.96 | 29,989 | 46.31% | 3073 | 2366 | 24,372 | 24,438 | 19,621 |
| *Stylonychia lemnae* ^f^ | 54.71 | 23,449 | 31.32% | 3089 | 2333 | 19,324 | 19,443 | 18,058 |
| *Tetmemena* sp. ^f^ | 60.63 | 25,219 | 37.00% | 3312 | 2404 | 18,718 | 18,166 | 16,577 |
| *Uronychia binucleata* ^e^ | 68.01 | 37,809 | 45.88% | 2318 | 1799 | 97 | 91 | 1 |
| *Uronychia setigera* ^e^ | 110.94 | 62,676 | 36.40% | 2553 | 1770 | 3,826 | 3,924 | 548 |
| *Urostyla* sp. ^f^ | 42.62 | 20,244 | 27.25% | 2898 | 2105 | 15,569 | 15,960 | 13,496 |
| ^a^ Chen et al. (2019); ^b^ Feng et al. (2022); ^c^ Zheng et al. (2021); ^d^ Swart et al. (2013); ^e^ Chen et al. (2021) (single-cell WGA data); ^f^ Chen et al. (2015); ^g^ Wang et al. (2016); ^h^ Li et al. (2021); ^i^ present study | | | | | | | | |

Table S1. Assembly features of spirotrichs.

Table S2. Two-gene chromosomes.

| Species | Trans- nanochomosomes | | Cis- nanochromosomes | |
| --- | --- | --- | --- | --- |
|  | count | percentage | count | percentage |
| *Strombidium* cf. *sulcutum* ^g^ | 50 | 6.0% | 777 | 94.0% |
| *Euplotes woodruffi* ^b^ | 495 | 10.7% | 4151 | 89.3% |
| *Euplotes octocarinatus* ^f^ | 373 | 23.5% | 1211 | 76.5% |
| *Euplotes vannus* ^a^ | 283 | 38.0% | 461 | 62.0% |
| *Tetmemena* sp. ^e^ | 1409 | 42.7% | 1889 | 57.3% |
| *Halteria.grandinella* ^c^ | 1191 | 51.1% | 1138 | 48.9% |
| *Oxytricha.trifallax* ^d^ | 1688 | 59.4% | 1152 | 40.6% |
| *Stylonychia lemnae* ^e^ | 1775 | 64.3% | 986 | 35.7% |
| ^a^ Chen et al. (2019); ^b^ Feng et al. (2022); ^c^ Zheng et al. (2021); ^d^ Swart et al. (2013);  ^e^ Chen et al. (2015); ^f^ Wang et al. (2016); ^g^ present study | | | | |

Table S3. TEs annotated in MIC assemblies in present study.

Table S4. The relative position of transcription start sites (TSS), transcription end sites (TES), start codons, stop codons, and AT-rich regions.

|  | ***E. vannus*** | ***O. trifallax*** | ***H. grandinella*** | ***S.* cf. *sulcatum*** |
| --- | --- | --- | --- | --- |
| **Single-gene chromosomes with both AT-rich regions (#)** | 20,064 | 12,116 | 3577 | 17,741 |
| **TSS located beyond AT-rich region (%)** | 75.6% | 90.6% | 91.3% | 81.1% |
| **TES located beyond AT-rich region (%)** | 56.4% | 51.8% | 96.8% | 81.5% |
| **Start codon located beyond AT-rich region (%)** | 92.6% | 99.6% | 91.3% | 85.5% |
| **Stop codon located beyond AT-rich region (%)** | 84.6% | 98.7% | 96.8% | 99.3% |

Table S5. Conserved “mobile IESs” annotated in present study.
